# Supplementary material for: Ultrasound renal denervation in hypertensive patients: A systematic review and meta-analysis
Source: PLoS One. 2025 Jan 15;20(1):e0311191. doi: 10.1371/journal.pone.0311191 (PMC11734901; doi:10.1371/journal.pone.0311191)
Supplement: S1 File — (1) Literature search terms, (2) PICOTS framework, (3A) list of examined studies, (3B) Demography and Clinical Characteristics of The Included Studies, (4) PRISMA Checklist, (5) Meta-regression Analysis of Gender on All Outcomes, and (6) Meta-regression Analysis of Sample Size on All Outcomes, and (7) Protocol for handling missing data. (DOCX) [file pone.0311191.s001.docx]

**SUPPLEMENTARY DATA 1.** **LITERATURE SEARCH TERMS**

| **Database** | **Keywords** |
| --- | --- |
| PubMed | #1 Ultrasound renal denervation [MeSH Terms]  #2 ((“ultrasound renal denervation”[Title/Abstract]) OR (“uRDN"[Title/Abstract]))  #3 #1 OR #2  #4 "Hypertension" [Title/Abstract])  #5 #2 AND #3, Filter : Full-text, Randomized Clinical Trial |
| Cochrane | #1 MeSH descriptor: [ultrasound renal denervation] explode all trees  #2 (“ultrasound renal denervation" OR “uRDN”):ti,ab,kw  #3 #1 OR #2  #4 ("hypertension"):ti,ab,kw  #5 #3 AND #4  #6 (“efficacy” OR effective* OR success*):ti,ab,kw  #7 #5 AND #6  #8 #7 AND (“trial”) |
| ScienceDirect | (“robotic-assisted percutaneous coronary intervention” OR “remote control percutaneous coronary intervention") AND ("safety" OR "overall death" OR "adverse events") AND ("efficacy" OR effective* OR success*) AND (“clinical trial”) |
| ProQuest | #1 mesh.Exact(“robotic-assisted percutaneous coronary intervention”)  #2 noft((“robotic-assisted percutaneous coronary intervention" OR “remote control percutaneous coronary intervention"))  #3 noft(("safety" OR “oil” OR “overall death” OR “adverse events”))  #4 noft(“efficacy” OR effective* OR success*)  #5 noft(“clinical trial”)  #6 (#1 OR #2) AND #3 AND #4 AND #5 |
| BMJ Journals | ((“Ultrasound renal denervation” OR “uRDN”) AND (“hypertension”) |
| Google Scholar | ((“Ultrasound renal denervation” OR “uRDN”) AND (“hypertension”) |
| Springer | ((“Ultrasound renal denervation” OR “uRDN”) AND (“hypertension”) |

**SUPPLEMENTARY DATA 2. PICOTS FRAMEWORK.**

| Patient | Hypertensive Patients |
| --- | --- |
| Intervention | Ultrasound Renal Denervation |
| Control | Hypertensive patients who received procedures other than Ultrasound Renal Denervation, such as angiography and renal angiogram. |
| Outcome | The Improvement of 24-hour Ambulatory Blood Pressure Monitoring, Daytime Ambulatory Blood Pressure Monitoring, Night-time Ambulatory Blood Pressure Monitoring, Home Ambulatory Blood Pressure Monitoring, and Office Ambulatory Blood Pressure Monitoring. |
| Time | Studies between 2017-2023 |
| Studies | Randomized Control Trial |

**SUPPLEMENTARY DATA 3A.** **A NUMBERED TABLE OF ALL STUDIES EXAMINED, INCLUDING THOSE THAT WERE EXCLUDED FROM THE ANALYSES**

| **No** | **Author, Year of Study** | **Title** | **Included in the Review** | **Reason of Exclusion** |
| --- | --- | --- | --- | --- |
|  | Azizi et al., 2023 | Endovascular Ultrasound Renal Denervation to Treat Hypertension: The RADIANCE II Randomized Clinical Trial | Yes | - |
|  | Azizi et al., 2018 | Endovascular ultrasound renal denervation to treat hypertension (RADIANCE-HTN SOLO): a multicentre, international, single-blind, randomised, sham-controlled trial | Yes | - |
|  | Kario et al., 2022 | Catheter-based ultrasound renal denervation in patients with resistant hypertension: the randomized, controlled REQUIRE trial | Yes | - |
|  | Mahfoud et al., 2021 | Changes in blood pressure after crossover to ultrasound renal denervation in patients initially treated with sham in the RADIANCE-HTN SOLO trial | Yes | - |
|  | Azizi et al., 2021 | Ultrasound renal denervation for hypertension resistant to a triple medication pill (RADIANCE-HTN TRIO): a randomised, multicentre, single-blind, sham-controlled trial | Yes | - |
|  | Azizi et al., 2019 | Six-Month Results of Treatment-Blinded Medication Titration for Hypertension Control After Randomization to Endovascular Ultrasound Renal Denervation or a Sham Procedure in the RADIANCE-HTN SOLO Trial | Yes | - |
|  | Fengler et al., 2017 | Three-Arm Randomized Trial of Different Renal Denervation Devices and Techniques in Patients With Resistant Hypertension (RADIOSOUND-HTN). | Yes | - |
|  | Saxena et al., 2022 | Predictors of blood pressure response to ultrasound renal denervation in the RADIANCE-HTN SOLO study | Yes |  |
|  | Azizi et al., 2020 | 12-Month Results From the Unblinded Phase of the RADIANCE-HTN SOLO Trial of Ultrasound Renal Denervation | No | Unblinded participant |
|  | Sanghvi et al., 2022 | Renal Artery Variations in Patients With Mild-to-Moderate Hypertension From the RADIANCE-HTN SOLO Trial | No | Different parameters |
|  | Azizi et al., 2019 | Six-Month Results of Treatment-Blinded Medication Titration for Hypertension Control After Randomization to Endovascular Ultrasound Renal Denervation or a Sham Procedure in the RADIANCE-HTN SOLO Trial | No | Different methods |
|  | Steinberg et al., 2020 | Effect of Renal Denervation and Catheter Ablation vs Catheter Ablation Alone on Atrial Fibrillation Recurrence Among Patients With Paroxysmal Atrial Fibrillation and Hypertension: The ERADICATE-AF Randomized Clinical Trial | No | Different interventions and comparison |
|  | Kandzari et al., 2018 | Effect of renal denervation on blood pressure in the presence of antihypertensive drugs: 6-month efficacy and safety results from the SPYRAL HTN-ON MED proof-of-concept randomised trial | No | Different methods |
|  | Worthley et al., 2017 | Safety and performance of the second generation EnligHTN™ Renal Denervation System in patients with drug-resistant, uncontrolled hypertension | No | Different intervention |
|  | Fisher et al., 2022 | Plasma renin and aldosterone concentrations related to endovascular ultrasound renal denervation in the RADIANCE-HTN SOLO trial | No | Different outcomes |
|  | Saxena et al., 2018 | Attenuation of Splanchnic Autotransfusion Following Noninvasive Ultrasound Renal Denervation: A Novel Marker of Procedural Success | No | Different outcome |
|  | Daemen et al., 2019 | Safety and efficacy of endovascular ultrasound renal denervation in resistant hypertension: 12-month results from the ACHIEVE study | No | Different study design |
| 18. | Pathak et al., 2023 | Alcohol-mediated renal denervation in patients with hypertension in the absence of antihypertensive medications | No | Different intervention |
| 19. | Elijovich et al., 2022 | Ultrasound renal denervation for hypertension: impact of the RADIANCE-HTN-TRIO trial on future management of resistant hypertension | No | No full-text available |
| 20. | Fengler et al., 2017 | Ultrasound-based renal sympathetic denervation for the treatment of therapy-resistant hypertension: a single-center experience | No | Different study design |
| 21. | Mauri et al.,, 2018 | A multinational clinical approach to assessing the effectiveness of catheter-based ultrasound renal denervation: The RADIANCE-HTN and REQUIRE clinical study designs | No | Different study design |
| 22. | Fengler et al., 2023 | 6- and 12-Month Follow-Up From a Randomized Clinical Trial of Ultrasound vs Radiofrequency Renal Denervation (RADIOSOUND-HTN) | No | Different comparison |
| 23. | Schneider et al., 2018 | Phase II randomized sham-controlled study of renal denervation for individuals with uncontrolled hypertension - WAVE IV | No | Different study design, no full-text available |

**SUPPLEMENTARY DATA 3B** **DEMOGRAPHY AND CLINICAL CHARACTERISTICS OF THE INCLUDED STUDIES**

| **Study** | **Year** | **Country** | **Age group (years)** | **Total patients (n)** | **Renal Denervation (n)** | **Sham (n)** | **Male (n)** | **Female (n)** | **Follow-up** | **Extracted Outcomes** | **Data Extractor** | **Date of Extraction** | **Confirmation of Data Eligibility** |
| --- | --- | --- | --- | --- | --- | --- | --- | --- | --- | --- | --- | --- | --- |
| Fengler et al., 2017 | 2017 | Europe | 63.9 ± 8.4 | 50 | 25 | 25 | NR | NR | 3 months | 24-h ambulatory, daytime ambulatory, night-time ambulatory, home ambulatory, office ambulatory | RNR | Feb 5, 2024 | Yes |
| Azizi et al., 2018 | 2018 | United States and Europe | 54.4 ± 10.2 | 146 | 74 | 72 | 85 | 61 | 3 years | 24-h ambulatory, daytime ambulatory, night-time ambulatory, home ambulatory, office ambulatory | RNR | Feb 5, 2024 | Yes |
| Azizi et al., 2019 | 2019 | United States and Europe | 54.1 ± 10.2 | 140 | 69 | 71 | 82 | 58 | 3 years | 24-h ambulatory, daytime ambulatory, night-time ambulatory, home ambulatory, office ambulatory | DDCHR | Feb 8, 2024 | Yes |
| Azizi et al., 2021 | 2021 | United States and Europe | 52.3 ± 7.5 | 136 | 69 | 67 | 109 | 27 | 3 years | 24-h ambulatory, daytime ambulatory, night-time ambulatory, home ambulatory, office ambulatory | DDCHR | Feb 8, 2024 | Yes |
| Mahfoud et al., 2021 | 2021 | United States and Europe | 54.1 ± 10.1,  54.0 ± 10.7 | 60 | 33 | 27 | 47 | 13 | 23 ± 6 months | 24-h ambulatory, daytime ambulatory, night-time ambulatory, office ambulatory | FAG | Feb 9, 2024 | Yes |
| Kario et al., 2021 | 2021 | Japan and South Korea | 50.7 ± 11.4 | 121 | 60 | 61 | 86 | 35 | 3 months | 24-h ambulatory, daytime ambulatory, night-time ambulatory, home ambulatory, office ambulatory | FAG | Feb 10, 2024 | Yes |
| Saxena et al., 2022 | 2022 | United States and Europe | 18-75 | 122 | 64 | 58 | NR | NR | 2 months | 24-h ambulatory, daytime ambulatory, night-time ambulatory, home ambulatory, office ambulatory | N | Feb 25, 2024 | Yes |
| Azizi et al., 2023 | 2023 | United States and Europe | 18-75 | 214 | 146 | 68 | NR | NR | 3 years | 24-h ambulatory, daytime ambulatory, night-time ambulatory, home ambulatory, office ambulatory | SEW | Feb 23, 2024 | Yes |

**SUPPLEMENTARY DATA 4.**  **PRISMA CHECKLIST**

| **Section and Topic** | **Item #** | **Checklist item** | **Location where item is reported** |
| --- | --- | --- | --- |
| **TITLE** | | |  |
| Title | 1 | Identify the report as a systematic review. | 1 |
| **ABSTRACT** | | |  |
| Abstract | 2 | See the PRISMA 2020 for Abstracts checklist. | 2 |
| **INTRODUCTION** | | |  |
| Rationale | 3 | Describe the rationale for the review in the context of existing knowledge. | 3-4 |
| Objectives | 4 | Provide an explicit statement of the objective(s) or question(s) the review addresses. | 4 |
| **METHODS** | | |  |
| Eligibility criteria | 5 | Specify the inclusion and exclusion criteria for the review and how studies were grouped for the syntheses. | 4-5 |
| Information sources | 6 | Specify all databases, registers, websites, organisations, reference lists and other sources searched or consulted to identify studies. Specify the date when each source was last searched or consulted. | 5 |
| Search strategy | 7 | Present the full search strategies for all databases, registers and websites, including any filters and limits used. | 5 |
| Selection process | 8 | Specify the methods used to decide whether a study met the inclusion criteria of the review, including how many reviewers screened each record and each report retrieved, whether they worked independently, and if applicable, details of automation tools used in the process. | 5 |
| Data collection process | 9 | Specify the methods used to collect data from reports, including how many reviewers collected data from each report, whether they worked independently, any processes for obtaining or confirming data from study investigators, and if applicable, details of automation tools used in the process. | 5 |
| Data items | 10a | List and define all outcomes for which data were sought. Specify whether all results that were compatible with each outcome domain in each study were sought (e.g. for all measures, time points, analyses), and if not, the methods used to decide which results to collect. | 5 |
|  | 10b | List and define all other variables for which data were sought (e.g. participant and intervention characteristics, funding sources). Describe any assumptions made about any missing or unclear information. | 5 |
| Study risk of bias assessment | 11 | Specify the methods used to assess risk of bias in the included studies, including details of the tool(s) used, how many reviewers assessed each study and whether they worked independently, and if applicable, details of automation tools used in the process. | 6 |
| Effect measures | 12 | Specify for each outcome the effect measure(s) (e.g. risk ratio, mean difference) used in the synthesis or presentation of results. | 6 |
| Synthesis methods | 13a | Describe the processes used to decide which studies were eligible for each synthesis (e.g. tabulating the study intervention characteristics and comparing against the planned groups for each synthesis (item #5)). | 6 |
|  | 13b | Describe any methods required to prepare the data for presentation or synthesis, such as handling of missing summary statistics, or data conversions. | 6 |
|  | 13c | Describe any methods used to tabulate or visually display results of individual studies and syntheses. | 6 |
|  | 13d | Describe any methods used to synthesize results and provide a rationale for the choice(s). If meta-analysis was performed, describe the model(s), method(s) to identify the presence and extent of statistical heterogeneity, and software package(s) used. | 6 |
|  | 13e | Describe any methods used to explore possible causes of heterogeneity among study results (e.g. subgroup analysis, meta-regression). | 6 |
|  | 13f | Describe any sensitivity analyses conducted to assess robustness of the synthesized results. | 6 |
| Reporting bias assessment | 14 | Describe any methods used to assess risk of bias due to missing results in a synthesis (arising from reporting biases). | 6 |
| Certainty assessment | 15 | Describe any methods used to assess certainty (or confidence) in the body of evidence for an outcome. | 6 |
| **RESULTS** | | |  |
| Study selection | 16a | Describe the results of the search and selection process, from the number of records identified in the search to the number of studies included in the review, ideally using a flow diagram. | 6-7 |
|  | 16b | Cite studies that might appear to meet the inclusion criteria, but which were excluded, and explain why they were excluded. | 6-7 |
| Study characteristics | 17 | Cite each included study and present its characteristics. | 7 |
| Risk of bias in studies | 18 | Present assessments of risk of bias for each included study. | 7 |
| Results of individual studies | 19 | For all outcomes, present, for each study: (a) summary statistics for each group (where appropriate) and (b) an effect estimate and its precision (e.g. confidence/credible interval), ideally using structured tables or plots. | 7-9 |
| Results of syntheses | 20a | For each synthesis, briefly summarise the characteristics and risk of bias among contributing studies. | 7-9 |
|  | 20b | Present results of all statistical syntheses conducted. If meta-analysis was done, present for each the summary estimate and its precision (e.g. confidence/credible interval) and measures of statistical heterogeneity. If comparing groups, describe the direction of the effect. | 7-9 |
|  | 20c | Present results of all investigations of possible causes of heterogeneity among study results. | 7-9 |
|  | 20d | Present results of all sensitivity analyses conducted to assess the robustness of the synthesized results. | 7-9 |
| Reporting biases | 21 | Present assessments of risk of bias due to missing results (arising from reporting biases) for each synthesis assessed. | 9 |
| Certainty of evidence | 22 | Present assessments of certainty (or confidence) in the body of evidence for each outcome assessed. | 9 |
| **DISCUSSION** | | |  |
| Discussion | 23a | Provide a general interpretation of the results in the context of other evidence. | 9-12 |
|  | 23b | Discuss any limitations of the evidence included in the review. | 13 |
|  | 23c | Discuss any limitations of the review processes used. | 13 |
|  | 23d | Discuss implications of the results for practice, policy, and future research. | 12-13 |
| **OTHER INFORMATION** | | |  |
| Registration and protocol | 24a | Provide registration information for the review, including register name and registration number, or state that the review was not registered. | 4 |
|  | 24b | Indicate where the review protocol can be accessed, or state that a protocol was not prepared. | 4 |
|  | 24c | Describe and explain any amendments to information provided at registration or in the protocol. | 4 |
| Support | 25 | Describe sources of financial or non-financial support for the review, and the role of the funders or sponsors in the review. | 14 |
| Competing interests | 26 | Declare any competing interests of review authors. | 14 |
| Availability of data, code and other materials | 27 | Report which of the following are publicly available and where they can be found: template data collection forms; data extracted from included studies; data used for all analyses; analytic code; any other materials used in the review. | 14 |

**SUPPLEMENTARY DATA 5. META-REGRESSION ANALYSIS OF GENDER ON ALL OUTCOMES**

*Supplementary Data 5.1. Meta-regression analysis on 24-h ambulatory blood pressure measurement.*

| Effect Size (β) | 0.034 |
| --- | --- |
| LCI | 0.015 |
| UCI | 0.053 |
| P value | < 0.001 |

Meta-regression analysis results, as seen in Supplementary Data 5.1., revealed that gender difference was associated with a significant increase in the risk of 24-hour ambulatory blood pressure measurement.

*Supplementary Data 5.2. Meta-regression analysis on daytime ambulatory blood pressure measurement.*

| Effect Size (β) | 0.021 |
| --- | --- |
| LCI | 0.005 |
| UCI | 0.047 |
| P value | 0.110 |

Meta-regression analysis results, as seen in **Supplementary Data 5.2.,** revealed that there was no statistically significant association between gender and daytime ambulatory blood pressure measurement.

*Supplementary Data 5.3. Meta-regression analysis on nighttime ambulatory blood pressure measurement.*

| Effect Size (β) | 0.140 |
| --- | --- |
| LCI | 0.085 |
| UCI | 0.169 |
| P value | < 0.001 |

Meta-regression analysis results, as seen in **Supplementary Data 5.3.,** revealed that gender was significantly associated with an increased risk of nighttime ambulatory blood pressure measurement.

*Supplementary Data 5.4. Meta-regression analysis on home ambulatory blood pressure measurement.*

| Effect Size (β) | 0.127 |
| --- | --- |
| LCI | 0.045 |
| UCI | 0.134 |
| P value | < 0.001 |

Meta-regression analysis results, as seen in **Supplementary Data 5.4.,** revealed that gender was significantly associated with an increased risk of home ambulatory blood pressure measurement.

*Supplementary Data 5.5. Meta-regression analysis on office ambulatory blood pressure measurement.*

| Effect Size (β) | 0.185 |
| --- | --- |
| LCI | 0.15 |
| UCI | 0.20 |
| P value | 0.081 |

Meta-regression analysis results, as seen in **Supplementary Data 5.5.,** revealed that gender was not significantly associated with an increased risk of office ambulatory blood pressure measurement.

**SUPPLEMENTARY DATA 6. META-REGRESSION ANALYSIS OF SAMPLE SIZE ON ALL OUTCOMES**

*Supplementary Data 6.1. Meta-regression analysis on 24-h ambulatory blood pressure measurement.*

| Effect Size (β) | 0.03 |
| --- | --- |
| LCI | 0.01 |
| UCI | 0.18 |
| P value | 0.0003 |

The meta-regression analysis result, as seen in **Supplementary Data 6.1.,** revealed that each one-unit increase in sample size was associated with a significant increase in the risk of 24-hour ambulatory blood pressure measurement.

Supplementary Data 6.2. Meta-regression analysis on daytime ambulatory blood pressure measurement.

| Effect Size (β) | 0.06 |
| --- | --- |
| LCI | 0.02 |
| UCI | 0.15 |
| P value | 0.007 |

Meta-regression analysis results, as seen in Table 2,s, as seen in **Supplementary Data 6.2.**, revealed that each one-unit increase in sample size was not associated with a significant increase in the risk of daytime ambulatory blood pressure measurement.

Supplementary Data 6.3. Meta-regression analysis on nighttime ambulatory blood pressure measurement.

| Effect Size (β) | 0.05 |
| --- | --- |
| LCI | 0.03 |
| UCI | 0.07 |
| P value | 0.25 |

Meta-regression analysis results, as seen in **Supplementary Data 6.3.**, revealed that sample size was not associated with increased risk of nighttime ambulatory blood pressure measurement.

Supplementary Data 6.4. Meta-regression analysis on home ambulatory blood pressure measurement

| Effect Size (β) | 0.09 |
| --- | --- |
| LCI | 0.04 |
| UCI | 0.12 |
| P value | < 0.005 |

Meta-regression analysis results, as seen in Supplementary Data 6.4., revealed that sample size was significantly associated with increased risk of home ambulatory blood pressure measurement.

Supplementary Data 6.5. Meta-regression analysis on office ambulatory blood pressure measurement.

| Effect Size (β) | 0.21 |
| --- | --- |
| LCI | 0.18 |
| UCI | 0.40 |
| P value | 0.0023 |

Meta-regression analysis results, as seen in **Supplementary Data 6.5.**, revealed that sample size was significantly associated with increased risk of office ambulatory blood pressure measurement.

**SUPPLEMENTARY DATA 7. PROTOCOL FOR HANDLING MISSING DATA**
In our study, missing data were handled through a systematic and transparent approach to ensure the robustness of our analyses. Specifically:

- - **Contacting Authors for Missing Data**: For studies where essential data were not reported, we reached out to the original authors via email to request the missing information. We allowed a reasonable timeframe for responses, typically 4-6 weeks. If we received no response, we proceeded with other methods of managing missing data.
  - **Imputation Methods**: In cases where missing data could not be obtained from the authors, we applied statistical imputation methods where appropriate. Depending on the nature of the missing data, we used methods such as:
  - **Mean Imputation**: For continuous variables missing from certain studies, we imputed the mean values derived from similar studies in the same subgroup or analysis.
  - **Sensitivity Analysis**: To assess the impact of the missing data on the overall results, we conducted sensitivity analyses by excluding studies with substantial missing data and compared the findings with the full dataset analysis. This allowed us to evaluate the potential bias introduced by missing information.
  - **Explicit Reporting of Missing Data**: We clearly noted in our manuscript and supplementary materials where data were missing and how it was managed. This includes flagging studies with missing variables, describing which imputation methods were used, and specifying the outcomes of the sensitivity analyses conducted to ensure the robustness of the final conclusions.
