# Supplementary material for: Ultrasound renal denervation in hypertensive patients: A systematic review and meta-analysis
Source: PLoS One. 2025 Jan 15;20(1):e0311191. doi: 10.1371/journal.pone.0311191 (PMC11734901; doi:10.1371/journal.pone.0311191)
Supplement: S2 File — (DOCX) [file pone.0311191.s002.docx]

**The Efficacy and Safety of Ultrasound Renal Denervation on Hypertensive Patients: A Meta-Analysis of Clinical Cases**

| **No** | **Author, Year of Study** | **Title** | **Included in the Review** | **Reason of Exclusion** |
| --- | --- | --- | --- | --- |
|  | Azizi et al., 2023 | Endovascular Ultrasound Renal Denervation to Treat Hypertension: The RADIANCE II Randomized Clinical Trial | Yes | - |
|  | Azizi et al., 2018 | Endovascular ultrasound renal denervation to treat hypertension (RADIANCE-HTN SOLO): a multicentre, international, single-blind, randomised, sham-controlled trial | Yes | - |
|  | Kario et al., 2022 | Catheter-based ultrasound renal denervation in patients with resistant hypertension: the randomized, controlled REQUIRE trial | Yes | - |
|  | Mahfoud et al., 2021 | Changes in blood pressure after crossover to ultrasound renal denervation in patients initially treated with sham in the RADIANCE-HTN SOLO trial | Yes | - |
|  | Azizi et al., 2021 | Ultrasound renal denervation for hypertension resistant to a triple medication pill (RADIANCE-HTN TRIO): a randomised, multicentre, single-blind, sham-controlled trial | Yes | - |
|  | Azizi et al., 2019 | Six-Month Results of Treatment-Blinded Medication Titration for Hypertension Control After Randomization to Endovascular Ultrasound Renal Denervation or a Sham Procedure in the RADIANCE-HTN SOLO Trial | Yes | - |
|  | Fengler et al., 2017 | Three-Arm Randomized Trial of Different Renal Denervation Devices and Techniques in Patients With Resistant Hypertension (RADIOSOUND-HTN). | Yes | - |
|  | Saxena et al., 2022 | Predictors of blood pressure response to ultrasound renal denervation in the RADIANCE-HTN SOLO study | Yes |  |
|  | Azizi et al., 2020 | 12-Month Results From the Unblinded Phase of the RADIANCE-HTN SOLO Trial of Ultrasound Renal Denervation | No | Unblinded participant |
|  | Sanghvi et al., 2022 | Renal Artery Variations in Patients With Mild-to-Moderate Hypertension From the RADIANCE-HTN SOLO Trial | No | Different parameters |
|  | Azizi et al., 2019 | Six-Month Results of Treatment-Blinded Medication Titration for Hypertension Control After Randomization to Endovascular Ultrasound Renal Denervation or a Sham Procedure in the RADIANCE-HTN SOLO Trial | No | Different methods |
|  | Steinberg et al., 2020 | Effect of Renal Denervation and Catheter Ablation vs Catheter Ablation Alone on Atrial Fibrillation Recurrence Among Patients With Paroxysmal Atrial Fibrillation and Hypertension: The ERADICATE-AF Randomized Clinical Trial | No | Different interventions and comparison |
|  | Kandzari et al., 2018 | Effect of renal denervation on blood pressure in the presence of antihypertensive drugs: 6-month efficacy and safety results from the SPYRAL HTN-ON MED proof-of-concept randomised trial | No | Different methods |
|  | Worthley et al., 2017 | Safety and performance of the second generation EnligHTN™ Renal Denervation System in patients with drug-resistant, uncontrolled hypertension | No | Different intervention |
|  | Fisher et al., 2022 | Plasma renin and aldosterone concentrations related to endovascular ultrasound renal denervation in the RADIANCE-HTN SOLO trial | No | Different outcomes |
|  | Saxena et al., 2018 | Attenuation of Splanchnic Autotransfusion Following Noninvasive Ultrasound Renal Denervation: A Novel Marker of Procedural Success | No | Different outcome |
|  | Daemen et al., 2019 | Safety and efficacy of endovascular ultrasound renal denervation in resistant hypertension: 12-month results from the ACHIEVE study | No | Different study design |
| 18. | Pathak et al., 2023 | Alcohol-mediated renal denervation in patients with hypertension in the absence of antihypertensive medications | No | Different intervention |
| 19. | Elijovich et al., 2022 | Ultrasound renal denervation for hypertension: impact of the RADIANCE-HTN-TRIO trial on future management of resistant hypertension | No | No full-text available |
| 20. | Fengler et al., 2017 | Ultrasound-based renal sympathetic denervation for the treatment of therapy-resistant hypertension: a single-center experience | No | Different study design |
| 21. | Mauri et al.,, 2018 | A multinational clinical approach to assessing the effectiveness of catheter-based ultrasound renal denervation: The RADIANCE-HTN and REQUIRE clinical study designs | No | Different study design |
| 22. | Fengler et al., 2023 | 6- and 12-Month Follow-Up From a Randomized Clinical Trial of Ultrasound vs Radiofrequency Renal Denervation (RADIOSOUND-HTN) | No | Different comparison |
| 23. | Schneider et al., 2018 | Phase II randomized sham-controlled study of renal denervation for individuals with uncontrolled hypertension - WAVE IV | No | Different study design, no full-text available |
| 24 | Bhatt et al., 2014 | A controlled trial of renal denervation for resistant hypertension | No | Different method employed |
| 25 | Fengler et al., 2019 | Renal Denervation in Isolated Systolic Hypertension Using Different Catheter Techniques and Technologies | No | Different comparator group |
| 26 | Neuzil et al., 2016 | Externally Delivered Focused Ultrasound for Renal Denervation | No | Different methods |
| 27 | Warchol-Celinska et al., 2018 | Renal Denervation in Resistant Hypertension and Obstructive Sleep Apnea: Randomized Proof-of-Concept Phase II Trial | No | Different population and intervention |
| 28 | Engholm et al., 2018 | Effects of renal denervation on coronary flow reserve and forearm dilation capacity in patients with treatment-resistant hypertension. A randomized, double-blinded, sham-controlled clinical trial | No | Different intervention |
| 29 | Ahmed et al., 2013 | Adjunctive renal sympathetic denervation to modify hypertension as upstream therapy in the treatment of atrial fibrillation (H-FIB) study: clinical background and study design | No | Protocol study |
| 30 | Rader et al., 2022 | Durability of blood pressure reduction after ultrasound renal denervation: three-year follow-up of the treatment arm of the randomised RADIANCE-HTN SOLO trial | No | Different methods (follow-up study) |
| 31 | Oliveras et al., 2018 | Organ damage changes in patients with resistant hypertension randomized to renal denervation or spironolactone: The DENERVHTA (Denervación en Hipertensión Arterial) study | No | Different intervention and comparator group |
| 32 | Chen et al., 2016 | The effect of two different renal denervation strategies on blood pressure in resistant hypertension: Comparison of full-length versus proximal renal artery ablation | No | Different methods |
| 33 | Verloop et al., 2013 | Renal denervation in heart failure with normal left ventricular ejection fraction. Rationale and design of the DIASTOLE (DenervatIon of the renAl Sympathetic nerves in hearT failure with nOrmal Lv Ejection fraction) trial | No | different methods |
| 34 | Courand et al., 2017 | Abdominal Aortic Calcifications Influences the Systemic and Renal Hemodynamic Response to Renal Denervation in the DENERHTN (Renal Denervation for Hypertension) Trial | No | Different methods, different intervention, and different comparator group |
| 35 | Azizi et al., 2022 | Effects of Renal Denervation vs Sham in Resistant Hypertension After Medication Escalation: Prespecified Analysis at 6 Months of the RADIANCE-HTN TRIO Randomized Clinical Trial | No | Different intervention |
| 36 | Rosa et al., 2016 | Role of Adding Spironolactone and Renal Denervation in True Resistant Hypertension: One-Year Outcomes of Randomized PRAGUE-15 Study | No | Different intervention |
| 37 | Kiuchi et al., 2018 | Pulmonary vein isolation combined with spironolactone or renal sympathetic denervation in patients with chronic kidney disease, uncontrolled hypertension, paroxysmal atrial fibrillation, and a pacemaker | No | Different methods, different intervention, different comparator |
| 38 | Lurz et al., 2020 | Changes in Stroke Volume After Renal Denervation: Insight From Cardiac Magnetic Resonance Imaging | No | Different intervention |
| 39 | Gosse et al., 2023 | Endovascular ultrasound renal denervation to lower blood pressure in young hypertensive women planning pregnancy: study protocol for a multicentre randomised, blinded and sham controlled proof of concept study | No | Protocol study |
| 40 | Liu et al,. 2017 | Efficacy and safety of renal denervation for Chinese patients with resistant hypertension using a microirrigated catheter: study design and protocol for a prospective multicentre randomised controlled trial | No | Different methods |
| 41 | Schonherr et al., 2016 | Retrospective morphometric study of the suitability of renal arteries for renal denervation according to the Symplicity HTN2 trial criteria | No | Different study design, different intervention, different methods, different outcomes |
| 42 | Ong et al., 2012 | Successful treatment of resistant hypertension with percutaneous renal denervation therapy | No | Different study design |
| 43 | Maclntyre IM,. et al 2019 | Renal denervation therapy for hypertension: still on trial | No | No full-text available, different study design |
| 44 | Amy et al., 2013 | 131 RENAL DENERVATION FOR RESISTANT HYPERTENSION: SAFETY AND RESPONSE RATE AT 6 MONTHS IN A UK POPULATION | No | No full-text available, different intervention |
| 45 | Kathoil et al., 2011 | Catheter-based renal sympathetic denervation reduces systolic blood pressure by 32 mm Hg in people with treatment-resistant hypertension | No | Different intervention |
| 46 | Ratcliffe et al., 2013 | 130 RENAL DENERVATION IMPROVES AUTONOMIC RESPONSES TO FALLS IN BLOOD PRESSURE | No | Different intervention |
| 47 | Hu et al., 2012 | EFFECTS OF RENAL SYMPATHETIC DENERVATION ON RATS WITH HEART FAILURE AFTER MYOCARDIAL INFARCTION | No | Different subject |
| 48 | Yuenhui et al., 2012 | TRANSCATHETER RENAL SYMPATHETIC MODIFICATION THERAPY FOR HEART FAILURE: PRIMARY EXPERIENCE OF RENAL DENERVATION THERAPY IN HEART FAILURE PATIENTS IN CHINA | No | Different intervention, different study design |
| 49 | Sharif et al,, 2020 | 16 Blood pressure reduction after catheter-based renal denervation in the absence of antihypertensive medications: spyral HTN-off med pivotal trial results | No | No full-text available, different intervention |
| 50 | Yang C et al., 2011 | Radiofrequency ablation for resistant hypertension: a report of three cases | No | Different study design |
| 51 | Williams et al., 2015 | Prevention And Treatment of Hypertension With Algorithm-based therapy (PATHWAY) number 2: protocol for a randomised crossover trial to determine optimal treatment for drug-resistant hypertension | No | Different study design, protocol study |
| 52 | Gassier JP et al., 2012 | The role of baroreflex activation therapy in sympathetic modulation for the treatment of resistant hypertension | No | Different study design |
| 53 | Bruce NS et al., 2011 | Novel stratified medicines approach to manage uncontrolled hypertension due to multiple drug intolerances | No | Different study design |
| 54 | Salam A et al., 2018 | Process evaluation of a randomised controlled trial of a pharmacological strategy to improve hypertension control: protocol for a qualitative study | No | Different study design, protocol study |
| 55 | Jalan et al., 1997 | Reduction in renal blood flow following acute increase in the portal pressure: evidence for the existence of a hepatorenal reflex in man? | No | Different study design |
| 56 | Matsuo Y et al., 2016 | Reduction in renal blood flow following acute increase in the portal pressure: evidence for the existence of a hepatorenal reflex in man? | No | Different study design |
| 57 | Chu et al., 2017 | American football and other sports injuries may cause migraine/persistent pain decades later and can be treated successfully with electrical twitch-obtaining intramuscular stimulation (ETOIMS) | No | Exclusion based on Title and abstract |
| 58 | Gut et al., 2005 | The hepatic sympathetic nerve plays a critical role in preventing Fas induced liver injury in mice | No | Exclusion based on title and abstract |
| 59 | Hurley et al., 2022 | Consensus practice guidelines on interventions for cervical spine (facet) joint pain from a multispecialty international working group | No | Exclusion based on title and abstract |
| 60 | Mathias., 1977 | Postural changes in plasma renin activity and responses to vasoactive drugs in a case of Shy-Drager syndrome. | No | Exclusion based on title and abstract |
| 61 | Eugene., 2019 | Ethnic differences in atrial fibrillation in patients with heart failure from Asia-Pacific | No | Exclusion based on title and abstract |
| 62 | Yu et al., 2019 | e0714 Autonomic nerve remodelling of prolonged rapid atrial pacing-induced atrial fibrillation and reversal effect of propranolol | No | Exclusion based on title and abstract |
| 63 | Misiewicz., 1974 | Pathophysiology of achalasia of the cardia | No | Exclusion based on title and abstract |
| 64 | Cattaneo ., 2006 | Pathological yawning as a presenting symptom of brain stem ischaemia in two patients | No | Exclusion based on title and abstract |
| 65 | Sakikabara et al., 2000 | Urinary dysfunction and orthostatic hypotension in multiple system atrophy: which is the more common and earlier manifestation? | No | Exclusion based on title and abstract |
| 66 | Yoneda et al., 2005 | Effect of central corticotropin releasing factor on hepatic circulation in rats: the role of the CRF2 receptor in the brain | No | Exclusion based on title and abstract |
| 67 | Rijnierse et al,, 2016 | Non-invasive imaging to identify susceptibility for ventricular arrhythmias in ischaemic left ventricular dysfunction | No | Exclusion based on title and abstract |
| 68 | Reffelmann et al., 2002 | Post-stenotic coronary blood flow at rest is not altered by therapeutic doses of the oral antidiabetic drug glibenclamide in patients with coronary artery disease | No | Exclusion based on title and abstract |
| 69 | Jones et al., 2012 | Safety and feasibility of hospital discharge 2 days following primary percutaneous intervention for ST-segment elevation myocardial infarction | No | Exclusion based on title and abstract |
| 70 | Maria et al., 2017 | Analgesic Effect and Functional Improvement Caused by Radiofrequency Treatment of Genicular Nerves in Patients With Advanced Osteoarthritis of the Knee Until 1 Year Following Treatment | No | Exclusion based on title and abstract |
| 71 | Han et al., 2021 | Brachial plexopathy as a complication of COVID-19 | No | Exclusion based on title and abstract |
| 72 | Yajnik et al., 2007 | 371 THE VALIDITY OF B-TYPE NATRIURETIC PEPTIDE BLOOD LEVELS TO DETECT HEART FAILURE IN HEART TRANSPLANT PATIENTS. | No | Exclusion based on title and abstract |
| 73 | Jieyu et al., 2013 | ASSA13-02-23 Relationship of Thickness of Left Atrial Epicardial Adipose Tissue and Atrial Fibrillation | No | Exclusion based on title and abstract |
| 74 | Herbert et al., 1975 | Natriuretic hormone—its possible role in fluid and electrolyte disturbances in chronic liver disease | No | Exclusion based on title and abstract |
| 75 | Middelorp et al., 2019 | Lifestyle modifications for treatment of atrial fibrillation | No | Exclusion based on title and abstract |
| 76 | Jenkins et al.,1997 | Vascular remodelling in intramyocardial resistance vessels in hypertensive human cardiac transplant recipients. | No | Exclusion based on title and abstract |
| 77 | Chockski et al., 2013 | Blood pressure and diabetes: vicious twins | No | Exclusion based on title and abstract |
| 78 | Xiaoyan et al., 2013 | ASSA13-02-21 Variations of Left Ventricular Conduction System and Markers of Successful Ablation in Patients with Verapamil–Sensitive Idiopathic Left Ventricular Tachycardia | No | Exclusion based on title and abstract |
| 79 | Jianqiang et al., 2010 | e0713 Evaluating successful ablation of scar-related atrial tachycardia originating at lateral wall of right atrium with a new method: strategic linear ablation to scar area isolation | No | Exclusion based on title and abstract |
| 80 | Liang et al., 2013 | GW24-e2374 Deficiency in Fractalkine Receptor Reduces Post-infarction Cardiac Rupture | No | Exclusion based on title and abstract |
| 81 | Shan et al., 2013 | GW24-e1356 The histocompatibility of angiotensin converting enzyme does not promote its function on inhibitory to negative ventricular remodelling | No | Exclusion based on title and abstract |
| 82 | Xiao et al., 2023 | Monitoring of the regulatory ability and regulatory state of the autonomic nervous system and its application to the management of hypertensive patients: a study protocol for randomised controlled trials | No | Protocol study, different methods |
| 83 | Charitakis et al., 2020 | Comparing efficacy and safety in catheter ablation strategies for atrial fibrillation: protocol of a network meta-analysis of randomised controlled trials | No | Protocol studies, different intervention |
| 84 | Townsend et al., 2018 | Catheter-Based Renal Denervation for Hypertension. | No | Different study design |
| 85 | Weber et al., 2019 | Renal Denervation for Treating Hypertension: Current Scientific and Clinical Evidence | No | Different study design |
| 86 | Fengler et al., 2022 | Renal Denervation for Resistant Hypertension: A Concise Update on Treatment Options and the Latest Clinical Evidence. | No | Different study design |
| 87 | Eleid et al., 2014 | Renal denervation for hypertension. | No | Different study design |
| 88 | Kirtane et al., 2023 | Patient-Level Pooled Analysis of Ultrasound Renal Denervation in the Sham-Controlled RADIANCE II, RADIANCE-HTN SOLO, and RADIANCE-HTN TRIO Trials | No | Different study design |
| 89 | Rao et al., 2022 | Update on Renal Sympathetic Denervation for the Treatment of Hypertension. | No | Different study design |
| 90 | Shah et al., 2022 | Effectiveness of renal denervation in the treatment of hypertension: a literature review. | No | Different study design |
| 91 | Gallo G., 2022 | [Resistant hypertension: integrated strategies between pharmacological treatment and renal denervation]. | No | Different study design,non-English literature |
| 92 | Haribabu et al., 2021 | Recent trends in renal denervation devices for resistant hypertension treatment. | No | Different study design |
| 93 | Korovesis et al., 2014 | Renal denervation for resistant hypertension: acute results and long-term follow-up. | No | Different study design, different intervention |
| 94 | Yap et al., 2021 | Renal Denervation in the treatment of Resistant Hypertension. | No | Different intervention |
| 95 | Froeschl et al., 2014 | Percutaneous renal sympathetic denervation: 2013 and beyond. | No | Different study design |
| 96 | Cao et al., 2021 | Renal denervation: a new therapeutic approach for resistant hypertension. | No | Different study design |
| 97 | Lauder et al., 2021 | The current status of renal denervation for the treatment of arterial hypertension. | No | Different study design |
| 98 | Schlaich et al., 2021 | Towards Establishing Renal Denervation as the Third Pillar in Hypertension Treatment: The RADIANCE-HTN TRIO Trial | No | Editorial comments |
| 99 | Scheurig-Muenkler C, et al., 2013 | Renal denervation for refractory hypertension - technical aspects, complications and radiation exposure. | No | Different study design |
| 100 | Kannan et al., 2014 | Renal sympathetic nervous system and the effects of denervation on renal arteries. | No | Different study design |
| 101 | Schmieder et al., 2016 | Non-invasive Renal Denervation: Update on External Ultrasound Approaches. | No | Different study design |
| 102 | Manukyan et al., 2022 | Favorable effect of renal denervation on elevated renal vascular resistance in patients with resistant hypertension and type 2 diabetes mellitus | No | Different study design, different intervention |
| 103 | Wang et al., 2022 | Renal Denervation Attenuates Adverse Remodeling and Intramyocardial Inflammation in Acute Myocardial Infarction With Ischemia-Reperfusion Injury. | No | Different study design |
| 104 | Bausback et al., 2014 | Renal denervation for hypertension refractory to renal artery stenting. | No | Different study design |
| 105 | Chernin et al., 2017 | Renal Sympathetic Denervation System via Intraluminal Ultrasonic Ablation: Therapeutic Intravascular Ultrasound Design and Preclinical Evaluation. | No | Different study design |
| 106 | Chen et al., 2019 | ReferencesEfficiency and safety of renal denervation via cryoablation (Cryo-RDN) in Chinese patients with uncontrolled hypertension: study protocol for a randomized controlled trial. | No | Different study design, protocol study |
| 107 | Mafoud et al., 2021 | Device Therapy of Hypertension. | No | Different study design |
| 108 | Mafoud et al., 2021 | Catheter-based alcohol-mediated renal denervation for the treatment of uncontrolled hypertension: design of two sham-controlled, randomized, blinded trials in the absence (TARGET BP OFF-MED) and presence (TARGET BP I) of antihypertensive medications. | No | Different intervention |
| 109 | Weber et al., 2021 | Improved Understanding of Renal Nerve Anatomy: An Opportunity to Enhance Denervation Treatment of Hypertension. | No | Different study design |
| 110 | Okamura et al., 2018 | The Prevalence of Japanese Outpatients with Hypertension Who Meet the Definition of Treatment Resistant Hypertension and Are Eligible for Enrolment in Clinical Trials of Endovascular Ultrasound Renal Denervation. | No | Different study design |
| 111 | Kjeldsen et al., 2018 | Renal denervation achieved by endovascular delivery of ultrasound in RADIANCE-HTN SOLO or by radiofrequency energy in SPYRAL HTN-OFF and SPYRAL-ON lowers blood pressure | No | Editorial, different comparison group |
| 112 | Rong et al., 2015 | Noninvasive Renal Denervation for Resistant Hypertension Using High-Intensity Focused Ultrasound | No | Different study design |
| 113 | Dimitriadis et al., 2020 | Renal denervation vascular effects: An "insider's" view. | No | Different study design |
| 114 | Fengler et al.., 2019 | A Three-Arm Randomized Trial of Different Renal Denervation Devices and Techniques in Patients With Resistant Hypertension (RADIOSOUND-HTN). | No | Duplicates |
| 115 | Yap et al., 2021 | Renal sympathetic denervation in the treatment of resistant hypertension. | No | Different intervention |
| 116 | Akinola., 1999 | Cardiovascular, autonomic, and plasma catecholamine responses in unilateral and bilateral carotid artery stenosis | No | Exclusion based on title and abstract |
| 117 | Ewen et al., 2016 | Anatomical and procedural determinants of catheter-based renal denervation. | No | Exclusion based on title and abstract, different intervention |
| 118 | Mabin et al., 2018 | First experience with endovascular ultrasound renal denervation for the treatment of resistant hypertension. | No | Exclusion based on title and abstract |
| 119 | Donazzan et al., 2016 | Effects of catheter-based renal denervation on cardiac sympathetic activity and innervation in patients with resistant hypertension. | No | Different intervention |
| 120 | Brandt et al., 2012 | Renal sympathetic denervation reduces left ventricular hypertrophy and improves cardiac function in patients with resistant hypertension. | No | Different study design |
| 121 | Fengler et al., 2017 | Blood Pressure Response to Main Renal Artery and Combined Main Renal Artery Plus Branch Renal Denervation in Patients With Resistant Hypertension. | No | Different study design, different intervention |
| 122 | Simonetti et al., 2012 | Endovascular radiofrequency renal denervation in treating refractory arterial hypertension: a preliminary experience. | No | Different study design, different intervention |
| 123 | Zhang et al., 2014 | The effects of catheter-based radiofrequency renal denervation on renal function and renal artery structure in patients with resistant hypertension. | No | Different study design, different intervention |
| 124 | Stiermaier et al., 2016 | Endovascular ultrasound for renal sympathetic denervation in patients with therapy-resistant hypertension not responding to radiofrequency renal sympathetic denervation. | No | Different intervention |
| 125 | Saxena et al., 2022 | Predictors of blood pressure response to ultrasound renal denervation in the RADIANCE-HTN SOLO study | No | Different method, different outcome |
| 126 | Hopper et al., 2017 | Sympathetic Response and Outcomes Following Renal Denervation in Patients With Chronic Heart Failure: 12-Month Outcomes From the Symplicity HF Feasibility Study. | No | Different intervention |
| 127 | Okamura et al., 2021 | Intravascular Ultrasound Can Be Used to Locate Nerves, but not Confirm Ablation, During Renal Sympathetic Denervation. | No | Different Methods |
| 128 | Budjan et al., 2016 | Renal Denervation in Patients with Resistant Hypertension-Assessment by 3T Renal 23Na-MRI: Preliminary Results. | No | Different study design |
| 129 | Baroni et al., 2015 | Effects of Renal Sympathetic Denervation on Arterial Stiffness and Blood Pressure Control in Resistant Hypertensive Patients: A Single Centre Prospective Study. | No | Different study design, different intervention, different outcome |
| 130 | Fezy et al., 2017 | Effect of catheter-based renal denervation on left ventricular function, mass and (un)twist with two-dimensional speckle tracking echocardiography. | No | Different intervention |
| 131 | Roleder et al., 2016 | Long-term follow-up of renal arteries after radio-frequency catheter-based denervation using optical coherence tomography and angiography. | No | Different study design |
| 132 | Yin et al., 2013 | ASSA13-02-25 CARTO Three-Dimensional Mapping System Guided Catheter Ablation of Macro-Reentrant Atrial Tachycardia Following Cardiac Surgery in Patients with Structural Heart Disease | No | Exclusion based on title and abstract |
| 133 | Jones et al., 2016 | Clinical challenges in the diagnosis and management of postural tachycardia syndrome | No | Exclusion based on title and abstract |
| 134 | Kushwaha et al., 1994 | Effect of beta blockade on the neurohumoral and cardiopulmonary response to dynamic exercise in cardiac transplant recipients. | No | Exclusion based on title and abstract |
| 135 | Hensch et al., 2010 | Cardiac neurotransmission imaging with 123I-meta-iodobenzylguanidine in postural tachycardia syndrome | No | Exclusion based on title and abstract |
| 136 | Naha et al., 2012 | Incidentally detected Monckeberg's sclerosis in a diabetic with coronary artery disease | No | Exclusion based on title and abstract |
| 137 | Yoshida et al., 2015 | Elevated plasma norepinephrine level and sick sinus syndrome in patients with lone atrial fibrillation | No | Exclusion based on title and abstract |
| 138 | Huihui et al., 2013 | ASSA13-02-26 Stroke in young patients with paroxysmal supraventricular tachycardia: A report of two cases of thromboembolic stroke | No | Exclusion based on title and abstract |
| 139 | Weng et al., 2017 | Migraine and subsequent chronic kidney disease risk: a nationwide population-based cohort study | No | Exclusion based on title and abstract |
| 140 | Humm et al., 2017 | Effects of water drinking on cardiovascular responses to supine exercise and on orthostatic hypotension after exercise in pure autonomic failure | No | Exclusion based on title and abstract |
| 141 | Spalding et al., 1969 | HIGH BLOOD PRESSURE | No | Exclusion based on title and abstract |
| 142 | Rimoldi et al., 2014 | Secondary arterial hypertension: when, who, and how to screen? | No | Exclusion based on title and abstract |
| 143 | Hoogerwaard et al., 2017 | Renal vascular calcification and response to renal nerve denervation in resistant hypertension. | No | Exclusion based on title and abstract |
| 144 | Ripp et al., 2015 | Predictors of Renal Denervation Efficacy in the Treatment of Resistant Hypertension. | No | Exclusion based on title and abstract |
| 145 | Alexander et al., 2014 | Renal denervation using focused infrared fiber lasers: a potential treatment for hypertension. | No | Exclusion based on title and abstract |
| 146 | Sorensen et al., 2014 | [Renal sympathetic denervation in treatment-resistant hypertension]. | No | Exclusion based on title and abstract |
| 147 | Krum et al., 2009 | Catheter-based renal sympathetic denervation for resistant hypertension: a multicentre safety and proof-of-principle cohort study. | No | Exclusion based on title and abstract |
| 148 | Lurz et al., 2019 | Lessons Learned from RADIOSOUND-HTN: Different Technologies and Techniques for Catheter-based Renal Denervation and Their Effect on Blood Pressure. | No | Exclusion based on title and abstract |
| 149 | Volz et al., 2019 | Effect of renal denervation on coronary flow reserve in patients with resistant hypertension. | No | Exclusion based on title and abstract |
| 150 | VonAchen et al., 2016 | Accessory renal arteries: Prevalence in resistant hypertension and an important role in nonresponse to radiofrequency renal denervation. | No | Exclusion based on title and abstract |
| 151 | Wang et al., 2018 | Effects of Renal Denervation on Cardiac Structural and Functional Abnormalities in Patients with Resistant Hypertension or Diastolic Dysfunction. | No | Different intervention, different outcome |
| 152 | Kiuchi et al., 2018 | Number of ablated spots in the course of renal sympathetic denervation in CKD patients with uncontrolled hypertension: EnligHTN vs. Standard irrigated cardiac ablation catheter. | No | Different intervention, different outcomes |
| 153 | Pua et al., 2016 | Safety and Feasibility of Renal Sympathetic Denervation in Patients With Insufficient Renal Artery Length. | No | Different intervention, different outcomes |
| 154 | Fengler et al., 2018 | Cardiac magnetic resonance assessment of central and peripheral vascular function in patients undergoing renal sympathetic denervation as predictor for blood pressure response. | No | Different intervention |
| 155 | Tsioufis et al., 2015 | Effects of multielectrode renal denervation on cardiac and neurohumoral adaptations in resistant hypertension with cardiac hypertrophy: an EnligHTN I substudy. | No | Different intervention, different outcome |
| 156 | Mahfoud et al., 2021 | Long-Term Results up to 12 Months After Catheter-Based Alcohol-Mediated Renal Denervation for Treatment of Resistant Hypertension. | No | Different intervention |
| 157 | Ott et al., 2012 | Renal denervation in a hypertensive patient with end-stage renal disease and small arteries: a direction for future research. | No | Exclusion based on title and abstract |
| 158 | Zeijen et al., 2023 | Unilateral renal atrophy 4 years after renal sympathetic denervation: a case report. | No | Different study design |
| 159 |  |  | No |  |
| 160 |  |  | No |  |
| 161 | Fengler et al., 2017 | Ultrasound-based renal sympathetic denervation for the treatment of therapy-resistant hypertension: a single-center experience | No | Different study design, duplicates |
| 162 | Waksman et al., 2013 | Beta radiation for renal nerve denervation: initial feasibility and safety. | No | Exclusion based on title and abstract |
| 163 | Delacroix et al., 2018 | Effects of renal sympathetic denervation on myocardial structure, function and perfusion: A serial CMR study. | No | Exclusion based on title and abstract |
| 164 | Neumann et al., 2016 | Effects of renal denervation on heart failure biomarkers and blood pressure in patients with resistant hypertension. | No | Exclusion based on title and abstract |
| 165 | Delgado-Sliva et al., 2022 | Risk Stratification and Cardiac Sympathetic Activity Assessment Using Myocardial [123I] MIBG Imaging in Renal Denervation. | No | Exclusion based on title and abstract |
| 166 | Skowerski et al., 2023 | Angio-computed tomography reveals differences in the anatomy of renal arteries in resistant hypertension patients qualified for renal denervation versus pseudo-resistant hypertensive subjects. | No | Exclusion based on title and abstract |
| 167 | Belardi et al., 2016 | Renal sympathetic denervation: Recalculating. | No | Exclusion based on title and abstract |
| 168 | Wang et al., 2013 | Noninvasive renal sympathetic denervation by extracorporeal high-intensity focused ultrasound in a pre-clinical canine model. | No | Exclusion based on title and abstract |
| 169 | Qingyan et al., 2015 | Beneficial Effects of Renal Denervation on Pulmonary Vascular Remodeling in Experimental Pulmonary Artery Hypertension. | No | Exclusion based on title and abstract |
| 170 | Bertog et al., 2021 | Alcohol-Mediated Renal Sympathetic Neurolysis for the Treatment of Hypertension: The Peregrine Infusion Catheter. | No | Exclusion based on title and abstract |
| 171 | Fischell et al., 2016 | Transcatheter Alcohol-Mediated Perivascular Renal Denervation With the Peregrine System: First-in-Human Experience. | No | Different study design |
| 172 | Zhang et al,. 2015 | Beneficial effects of renal denervation on pulmonary vascular remodeling in experimental pulmonary artery hypertension | No | Different study design, non-English literature |
| 173 | Kiuchi et al., 2017 | Acute Vasodilation Caused by Different Strategies of Renal | No |  |
| 174 | Trabattoni et al., 2017 | Renal denervation in a patient with unilateral ectopic kidney in the pelvis and refractory hypertension. | No | Exclusion based on title and abstract |
| 175 | Cohen et al., 2021 | Imaging strategies for safety surveillance after renal artery denervation. | No | Exclusion based on title and abstract |
| 176 | Li et al., 2015 | Renal denervation using catheter-based radiofrequency ablation with temperature control: renovascular safety profile and underlying mechanisms in a hypertensive canine model. | No | Exclusion based on title and abstract |
| 177 | Ribichini et al., 2013 | Invasive assessment of renal artery atherosclerotic disease and resistant hypertension before renal sympathetic denervation. | No | Exclusion based on title and abstract |
| 178 | Kresoja et al., 2021 | Renal Sympathetic Denervation in Patients With Heart Failure With Preserved Ejection Fraction. | No | Exclusion based on title and abstract |
| 179 | Han et al., 2020 | The safety of renal denervation as assessed by optical coherence tomography: pre- and post-procedure comparison with multi-electrode ablation catheter in animal experiment. | No | Exclusion based on title and abstract |
| 180 | Rippy et al., 2011 | Catheter-based renal sympathetic denervation: chronic preclinical evidence for renal artery safety. | No | Different study design |
| 181 | Lauder et al., 2018 | Anatomical and procedural determinants of ambulatory blood pressure lowering following catheter-based renal denervation using radiofrequency. | No | Exclusion based on title and abstract |
| 182 | Ewen et al., 2013 | Percutaneous renal denervation: new treatment option for resistant hypertension and more? | No | Exclusion based on title and abstract |
| 183 | Hearon et al., 2021 | Evidence of Reduced Efferent Renal Sympathetic Innervation After Chemical Renal Denervation in Humans. | No | Exclusion based on title and abstract |
| 184 | Voora et al,.2018 | Modulation of Sympathetic Overactivity to Treat Resistant Hypertension. | No | Exclusion based on title and abstract |
| 185 | Karanasos et al., 2015 | Multimodality Intra-Arterial Imaging Assessment of the Vascular Trauma Induced by Balloon-Based and Nonballoon-Based Renal Denervation Systems. | No | Exclusion based on title and abstract |
| 186 | Kario et al., 2023 | Anti-hypertensive medication adherence in the REQUIRE trial: post-hoc exploratory evaluation. | No | Exclusion based on title and abstract |
| 187 | Sakakura et al., 2015 | Controlled circumferential renal sympathetic denervation with preservation of the renal arterial wall using intraluminal ultrasound: a next-generation approach for treating sympathetic overactivity. | No | Exclusion based on title and abstract |
| 188 | Rimoldi et al., 2015 | Resistant hypertension: what the cardiologist needs to know. | No | Exclusion based on title and abstract |
| 189 | Chermin et al., 2018 | First-in-Man Experience with a Novel Catheter-Based Renal Denervation System of Ultrasonic Ablation in Patients with Resistant Hypertension. | No | Exclusion based on title and abstract |
| 190 | Shea et al., 2017 | Effect of applied energy in renal sympathetic denervation with magnetic resonance guided focused ultrasound in a porcine model. | No | Exclusion based on title and abstract |
| 191 | Singh et al., 2019 | Sustained Decrease in Blood Pressure and Reduced Anatomical and Functional Reinnervation of Renal Nerves in Hypertensive Sheep 30 Months After Catheter-Based Renal Denervation. | No | Exclusion based on title and abstract |
| 192 | Rimoldi et al., 2014 | Anatomical eligibility of the renal vasculature for catheter-based renal denervation in hypertensive patients. | No | Exclusion based on title and abstract |
| 193 | Lorelius et al., 1978 | Renal haemodynamics before and after splanchnic block in patients with hypertension. | No | Exclusion based on title and abstract |
| 194 | Schmid et al., 2016 | Mid-Term Vascular Safety of Renal Denervation Assessed by Follow-up MR Imaging. | No | Different study design, different intervention, different outcome |
| 195 | Liuchi et al., 2019 | New Approaches in the Management of Sudden Cardiac Death in Patients with Heart Failure-Targeting the Sympathetic Nervous System. | No | Exclusion based on title and abstract |
| 196 | Ziegler et al., 2013 | Renal denervation in a patient with prior renal artery stenting. | No | Different study design |
| 197 | Song et al., 2020 | Quantitative analysis of renal arterial variations affecting the eligibility of catheter-based renal denervation using multi-detector computed tomography angiography. | No | Different intervention |
| 198 | Palionis et al., 2016 | Could careful patient selection for renal denervation warrant a positive effect on arterial stiffness and left ventricular mass reduction? | No | Exclusion based on title and abstract |
| 199 | Polhemus et al., 2017 | Renal Sympathetic Denervation Protects the Failing Heart Via Inhibition of Neprilysin Activity in the Kidney. | No | Exclusion based on title and abstract |
| 200 | Yang et al., 2013 | Percutaneous renal sympathetic denervation for the treatment of resistant hypertension with heart failure: first experience in Korea. | No | Different study design |
| 201 | Marshall et al., 2014 | Interventional radiologic techniques for screening, diagnosis and treatment of patients with renal artery stenosis. | No | Exclusion based on title and abstract |
| 202 | Spinelli et al., 2014 | Technical aspects of renal denervation in end-stage renal disease patients with challenging anatomy. | No | Exclusion based on title and abstract |
| 203 | White et al., 2021 | Renal Arteries Revisited: Anatomy, Pathologic Entities, and Implications for Endovascular Management. | No | Exclusion based on title and abstract |
| 204 | Freyhardt et al., 2018n | Renal Denervation by Transaortic Periarterial Ethanol Injection: An Experimental Study in Porcines. | No | Exclusion based on title and abstract |
| 205 | Verloop et al., 2015 | Effects of renal denervation on end organ damage in hypertensive patients | No | Exclusion based on title and abstract |
| 206 | Rundqvist et al., 2013 | Catheter-based renal denervation: a new method for resistant hypertension. Initial experiences of the procedure shows significant decrease in blood pressure. | No | Different intervention |
| 207 | Honton et al., 2014 | First report of transradial renal denervation with the dedicated radiofrequency Iberis catheter. | No | Different intervention |
| 208 | Castelejin et al., 2014 | Chronic kidney pain in autosomal dominant polycystic kidney disease: a case report of successful treatment by catheter-based renal denervation. | No | Different study design |
| 209 | Okamura et al., 2019 | A Case of Primary Aldosteronism Due to A Primary Adrenal Adenoma Diagnosed by Segmental Adrenal Venous Sampling (S-AVS) Using a Modified Catheter System and Lateral Cine Angiography. | No | Different study design |
| 210 | Kaczmarek et al., 2013 | An electrophysiological approach using 3D electroanatomical mapping system for catheter-based renal denervation: the first Polish experience. | No | Different study design |
| 211 | Rosenbaum et al., 2012 | An abdominal CT scan in first-line is an efficient investigation of uncontrolled hypertensives suspected to have an adrenal cause. | No | Exclusion based on title and abstract |
| 212 | Giordano et al., 2014 | Transcatheter renal sympathetic denervation despite angiographically significant proximal stenosis: proof of concept from a case report. | No | Different study design |
| 213 | Azizi et al., 2021 | Ultrasound renal denervation for hypertension resistant to a triple medication pill (RADIANCE-HTN TRIO): a randomised, multicentre, single-blind, sham-controlled trial | No | Duplicate study |
| 214 | Kirtane et al., 2022 | Cost-Effectiveness of Ultrasound Renal Denervation in Patients With Resistant Hypertension | No | Different outcome, no full-text |
| 215 | Elijovich., 2022 | Ultrasound renal denervation for hypertension: impact of the RADIANCE-HTN-TRIO trial on future management of resistant hypertension | No | Different study design |
| 216 | Schmieder et al., 2015 | TCT-87 Non-invasive Renal Denervation Study Using Externally Delivered Focused Ultrasound in Severe Resistant Hypertension: 1 Year Follow up Results | No | Different study design |
| 217 | Schmieder et al., 2016 | PREDICTORS OF BLOOD PRESSURE RESPONSE IN THE NON-INVASIVE RENAL DENERVATION STUDY USING EXTERNALLY DELIVERED FOCUSED ULTRASOUND IN SEVERE RESISTANT HYPERTENSION | No | No full-text available |
| 218 | Ray-Garcia et al., 2022 | Renal Denervation: A Review | No | Exclusion based on title and abstract |
| 219 | Townsend., 2019 | Treating Hypertension Using Renal Artery Denervation: Problems and Progress | No | Exclusion based on title and abstract |
| 220 | Tannu et al., 2023 | The Evolution of Pulmonary Artery Denervation for Treatment of Pulmonary Arterial Hypertension | No | Exclusion based on title and abstract |
| 221 | Guber et al., 2022 | Renal Sympathetic Denervation for Hypertension | No | Exclusion based on title and abstract |
| 222 | Coates et al., 2022 | Time, Temperature, Power, and Impedance Considerations for Radiofrequency Catheter Renal Denervation | No | Exclusion based on title and abstract |
| 223 | Liu et al., 2023 | New trends in non-pharmacological approaches for cardiovascular disease: Therapeutic ultrasound | No | Exclusion based on title and abstract |
| 224 | Ghorani et al., 2022 | Arterial hypertension – Clinical trials update 2021 | No | Exclusion based on title and abstract |
| 225 | Hyder et al., 2022 | Percutaneous Treatments for Pulmonary Hypertension: Reviewing the Growing Procedural Role for Interventional Cardiology | No | Exclusion based on title and abstract |
| 226 | Nunez-Gil et al., 2022 | Device-Based Therapies for Resistant Hypertension: Implications for the Perioperative Clinician | No | Exclusion based on title and abstract |
| 227 | Lao., 2022 | The obstetric implications of pulmonary hypertension and lung transplant | No | Exclusion based on title and abstract |
| 228 | Rebecca te al., 2023 | Multimodality Imaging of Right Heart Function: JACC Scientific Statement | No | Exclusion based on title and abstract |
| 229 | Hewson et al., 2023 | Postoperative ulnar neuropathy: a systematic review of evidence with narrative synthesis | No | Exclusion based on title and abstract |
| 230 | Squires et al., 2022 | Cardiac rehabilitation for heart transplant patients: Considerations for exercise training | No | Exclusion based on title and abstract |
| 231 | Bamfurd et al., 2022 | A State-of-the-Art Review: The Percutaneous Treatment of Highly Calcified Lesions | No | Exclusion based on title and abstract |
| 232 | Lauder et al., 2021 | The current status of renal denervation for the treatment of arterial hypertension | No | Different study design |
| 233 | Gonzales et al., 2022 | Paraneoplastic syndromes review: The great forgotten ones | No | Different study design |
| 234 | Townsend et al., 2019 | Treating Hypertension Using Renal Artery Denervation: Problems and Progress | No | Different study design |
| 235 | Papademitriou et al., 2019 | New data, new studies, new hopes for renal denervation in patients with uncontrolled hypertension | No | Different study design |
| 236 | Bardill et al., 2022 | Topical gel-based biomaterials for the treatment of diabetic foot ulcers | No | Exclusion based on title and abstract |
| 237 | Karam et al., 2023 | Approach to Diagnosis and Management of Hypertension: A Comprehensive and Combined Pediatric and Adult Perspective | No | Exclusion based on title and abstract |
| 238 | Alkhouli et al., 2022 | The Heart Brain Team and Patient-Centered Management of Ischemic Stroke | No | Exclusion based on title and abstract |
| 239 | Madeline et al., 2023 | Neuroimmune interplay in kidney health and disease: Role of renal nerves | No | Exclusion based on title and abstract |
| 240 | Liu et al., 2023 | New trends in non-pharmacological approaches for cardiovascular disease: Therapeutic ultrasound | No | Exclusion based on title and abstract |
| 241 | Kim et al., 2023 | Renal Transplantation: Immediate and Late Complications | No | Exclusion based on title and abstract |
| 242 | Delcroix et al., 2023 | Chronic thromboembolic pulmonary hypertension: realising the potential of multimodal management | No | Exclusion based on title and abstract |
| 243 | Pahuja et al.,2023 | Neuromodulation Therapies in Heart Failure: A State-of-the-Art Review | No | Exclusion based on title and abstract |
| 244 | Davenport., 2023 | Why is Intradialytic Hypotension the Commonest Complication of Outpatient Dialysis Treatments? | No | Exclusion based on title and abstract |
| 245 | Wang et al., 2023 | Chronic kidney disease-induced muscle atrophy: Molecular mechanisms and promising therapies | No | Exclusion based on title and abstract |
| 246 | Khatsgir., 2022 | Assessment of lower urinary tract symptoms | No | Exclusion based on title and abstract |
| 247 | Velasco et al., 2023 | Beyond conventional physical examination in hepatology: POCUS | No | Exclusion based on title and abstract |
| 248 | Forde et al., 2022 | Management of Biliary Complications in Liver Transplant Recipients | No | Exclusion based on title and abstract |
| 249 | Grosso et al., 2022 | Current treatment options and novel nanotechnology-driven enzyme replacement strategies for lysosomal storage disorders | No | Exclusion based on title and abstract |
| 250 | Weber et al., 2019 | Renal Denervation for Treating Hypertension: Current Scientific and Clinical Evidence | No | Different study design, different intervention |
| 251 | Gupta et al., 2020 | Renal denervation: Alternative treatment options for hypertension? | No | Different study design, different intervention |
| 252 | Adeyeye et al., 2022 | Hypertension | No | Exclusion based on title and abstract |
| 253 | Ott et al., 2022 | Diagnosis and treatment of arterial hypertension 2021 | No | Exclusion based on title and abstract |
| 254 | Zafeiropoulus et al., 2022 | Autonomic Neuromodulation for Atrial Fibrillation Following Cardiac Surgery: JACC Review Topic of the Week | No | Exclusion based on title and abstract |
| 255 | Raisi et al., 2020 | Renal Artery Denervation in Resistant Hypertension: The Good, The Bad and The Future | No | Different study design, different intervention |
| 256 | Lee et al., 2020 | Renal Denervation: History and Current Status | No | Different study design |
| 257 | Shidharan et al., 2022 | Autonomic nervous system and arrhythmias in structural heart disease | No | Exclusion based on title and abstract |
| 258 | Akhouli et al., 2022 | The Heart Brain Team and Patient-Centered Management of Ischemic Stroke | No | Exclusion based on title and abstract |
| 259 | Atreya et al., 2022 | Best Practices for the Catheter Ablation of Ventricular Arrhythmias | No | Exclusion based on title and abstract |
| 260 | Oluwasaeun et al., 2021 | Renal Sympathetic Denervation: A Comprehensive Review | No | Different study design, different intervention |
| 261 | Hoogerward et al., 2020 | Is renal denervation still a treatment option in cardiovascular disease? | No | Different study design, different intervention |
| 262 | Bartog et al., 2021 | Alcohol-Mediated Renal Sympathetic Neurolysis for the Treatment of Hypertension: The Peregrine™ Infusion Catheter | No | Exclusion based on title and abstract |
| 263 | Constantine et al., 2021 | Pulmonary artery denervation for pulmonary arterial hypertension | No | Exclusion based on title and abstract |
| 264 | Ferdinand et al., 2020 | The NEW-HOPE study and emerging therapies for difficult-to-control and resistant hypertension | No | Exclusion based on title and abstract |
| 265 | Brouwers et al., 2021 | Arterial hypertension | No | Exclusion based on title and abstract |
| 266 | Carey.,2020 | Special Article - The management of resistant hypertension: A 2020 update | No | Exclusion based on title and abstract |
| 267 | Delalio et al,, 2020 | Sympathetic Nervous System Contributions to Hypertension: Updates and Therapeutic Relevance | No | Exclusion based on title and abstract |
| 268 | Romero et al., 2021 | Advanced Therapies for Ventricular Arrhythmias in Patients With Chagasic Cardiomyopathy: JACC State-of-the-Art Review | No | Exclusion based on title and abstract |
| 269 | Wang et al., 2019 | Ablation of Neuroaxial in Patients with Ventricular Tachycardia | No | Exclusion based on title and abstract |
| 270 | Medranda et al., 2019 | Review of PCR e-Course 2020 Late-Breaking Clinical Trials | No | Exclusion based on title and abstract |
| 271 | Sen et al,. 2019 | Complications of Solid Organ Transplantation: Cardiovascular, Neurologic, Renal, and Gastrointestinal | No | Exclusion based on title and abstract |
| 272 | Ozemek et al., 2020 | Impact of therapeutic lifestyle changes in resistant hypertension | No | Exclusion based on title and abstract |
| 273 | Gatz et al., 2020 | Evaluation of the Renal Transplant Recipient in the Emergency Department | No | Exclusion based on title and abstract |
| 274 | Tanaka et al., 2019 | AKI and the Neuroimmune Axis | No | Exclusion based on title and abstract |
| 275 | Tanaka et al., 2020 | Crosstalk between the nervous system and the kidney | No | Exclusion based on title and abstract |
| 276 | Yildiz et al., 2020 | Left ventricular hypertrophy and hypertension | No | Exclusion based on title and abstract |
| 277 | Israel et al., 2013 | Sympathetic renal denervation: Hypertension beyond SYMPLICITY | No | Different study design |
| 278 | Zhong et al., 2017 | Renal Denervation to Modify Hypertension and the Heart Failure State | No | Different study design |
| 279 | Laffin et al., 2015 | Renal Denervation for Resistant Hypertension and Beyond | No | Different study design |
| 280 | Wasnik et al., 2021 | Multimodality imaging of pancreas-kidney transplants | No | Exclusion based on title and abstract |
| 281 | Morganti et al., 2018 | Resistant hypertension: Renal denervation or intensified medical treatment? | No | Exclusion based on title and abstract |
| 282 | Reilly et al., 2016 | Renal Denervation for Resistant Hypertension | No | Exclusion based on title and abstract |
| 283 | Stoiffer et al., 2013 | Catheter-based renal denervation in the treatment of resistant hypertension | No | Different study design, different intervention |
| 284 | Theodorakopolou et al., 2019 | Hypoxia and Endothelial Dysfunction in Autosomal-Dominant Polycystic Kidney Disease | No | Exclusion based on title and abstract |
| 285 | Zhu et al., 2019 | Neuromodulation for Ventricular Tachycardia and Atrial Fibrillation: A Clinical Scenario-Based Review | No | Exclusion based on title and abstract |
| 286 | Esler et al., 2017 | The future of renal denervation | No | Different study design |
| 287 | Xu et al., 2013 | Renal denervation: current implications and future perspectives | No | Different study design |
| 288 | Epstein et al., 2015 | Is the failure of SYMPLICITY HTN-3 trial to meet its efficacy endpoint the “end of the road” for renal denervation? | No | Different study design, different outcome |
| 289 | Koralata et al., 2019 | The Nephrologist as an Ultrasonographer | No | Exclusion based on title and abstract |
| 290 | Waldron et al., 2019 | Neuromodulation for the Treatment of Heart Rhythm Disorders | No | Exclusion based on title and abstract |
| 291 | Olsen et al., 2015 | Renal denervation | No | Different study design |
| 292 | Rothstein et al., 2013 | Commentary: Renal Nerve Denervation: Is Renervation an Issue? | No | Different study design |
| 293 | Oppenheimer et al., 2019 | Sonography of Acute Cholecystitis and Its Mimics | No | Exclusion based on title and abstract |
| 294 | Tena et al., 2020 | Perioperative considerations for kidney and pancreas-kidney transplantation | No | Exclusion based on title and abstract |
| 295 | Long et al., 2021 | A primer for managing cardiac transplant patients in the emergency department setting | No | Exclusion based on title and abstract |
| 296 | Elsokkari et al.,2021 | Electrical storm: Prognosis and management | No | Exclusion based on title and abstract |
| 297 | Shapiro et al., 2019 | Preventive Cardiology as a Subspecialty of Cardiovascular Medicine: JACC Council Perspectives | No | Exclusion based on title and abstract |
| 298 | Neethling et al., 2020 | Intraoperative and Early Postoperative Management of Heart Transplantation: Anesthetic Implications | No | Exclusion based on title and abstract |
| 299 | Chow et al.,2021 | Clinical presentation and management of myocardial infarction with nonobstructive coronary arteries (MINOCA): A literature review | No | Exclusion based on title and abstract |
| 300 | Saad et al., 2020 | Evidence-based pharmacotherapy for prevention and management of cardiac allograft vasculopathy | No | Exclusion based on title and abstract |
| 301 | Sakakura et al., 2014 | Methodological Standardization for the Pre-Clinical Evaluation of Renal Sympathetic Denervation | No | Different study design, different outcome |
| 302 | Gulati et al., 2013 | Review of the state of renal nerve ablation for patients with severe and resistant hypertension | No | Different study design |
| 303 | Tellez et al., 2016 | A Perspective on the Delivery of Renal Denervation Therapy Based on Pre-Clinical Data | No | Different study design, different outcome |
| 304 | Vemulapalli et al., 2014 | Apparent Treatment-Resistant Hypertension and Chronic Kidney Disease: Another Cardiovascular-Renal Syndrome? | No | Exclusion based on title and abstract |
| 305 | Smith et al., 2016 | Management of Renovascular Hypertension | No | Exclusion based on title and abstract |
| 306 | Rossignol et al., 2017 | Review article  The double challenge of resistant hypertension and chronic kidney disease | No | Exclusion based on title and abstract |
| 307 | Esler., 2014 | Illusions of truths in the Symplicity HTN-3 trial: generic design strengths but neuroscience failings | No | Exclusion based on title and abstract |
| 308 | Courand et al., 2014 | Baroreceptor stimulation for resistant hypertension: First implantation in France and literature review | No | Exclusion based on title and abstract |
| 309 | Schiller et al., 2017 | Eppur Si Muove: The dynamic nature of physiological control of renal blood flow by the renal sympathetic nerves | No | Exclusion based on title and abstract |
| 310 | Mousa et al., 2013 | Renal fibromuscular dysplasia | No | Exclusion based on title and abstract |
| 311 | Lakkis et al., 2014 | Treatment-Resistant Hypertension in the Transplant Recipient | No | Exclusion based on title and abstract |
| 312 | Grenier et al., 2013 | Ultrasound Elastography of the Kidney | No | Exclusion based on title and abstract |
| 313 | Anyaegbu et al., 2014 | Hypertension in the Teenager | No | Exclusion based on title and abstract |
| 314 | Chatterjee et al., 2015 | Novel Interventional Therapies to Modulate the Autonomic Tone in Heart Failure | No | Exclusion based on title and abstract |
| 315 | Bhramra-Ariza et al., 2014 | Percutaneous Interventional Therapies for the Treatment of Patients With Severe Pulmonary Hypertension | No | Exclusion based on title and abstract |
| 316 | Rodgers et al,, 2014 | Ultrasonographic Evaluation of the Renal Transplant | No | Exclusion based on title and abstract |
| 317 | Gonzales-Aguirre et al., 2016 | Managing Complications Following Nephron-Sparing Procedures for Renal Masses | No | Exclusion based on title and abstract |
| 318 | Inci et al., 2014 | Renal Transplant Complications: Diagnostic and Therapeutic Role of Radiology | No | Exclusion based on title and abstract |
| 319 | Giuliani et al., 2013 | Anorectal malformation and associated end-stage renal disease: Management from newborn to adult life | No | Exclusion based on title and abstract |
| 320 | Boor et al., 2015 | Renal Allograft Fibrosis: Biology and Therapeutic Targets | No | Exclusion based on title and abstract |
| 321 | Bunte et al., 2013 | Endovascular Treatment of Resistant and Uncontrolled Hypertension: Therapies on the Horizon | No | Different study design |
| 322 | Eirin et al., 2016 | Emerging concepts for patients with treatment-resistant hypertension | No | Different study design, different outcome |
| 323 | Tellman et al., 2015 | Management of Pain in Autosomal Dominant Polycystic Kidney Disease and Anatomy of Renal Innervation | No | Different study design, different outcome |
| 324 | Wang et al., 2013 | Noninvasive Renal Sympathetic Denervation by Extracorporeal High-Intensity Focused Ultrasound in a Pre-Clinical Canine Model | No | Excluded based on title and abstract |
| 325 | Eleid et al., 2014 | Renal Denervation for Hypertension | No | Different intervention |
| 326 | Worthley et al., 2015 | Safety and performance of the next generation EnligHTN™ renal denervation system in patients with drug-resistant, uncontrolled hypertension: The EnligHTN III first-in-human multicentre study | No | Different intervention |
| 327 | Almeida et al., 2015 | Renal denervation for resistant hypertension | No | Different intervention |
| 328 | Vasa et al., 2015 | An update of the expert consensus statement of the Czech Hypertension Society on renal denervation in resistant hypertension | No | Exclusion based on title and abstract |
| 329 | Stacnecsu et al., 2016 | Pediatric Abdominal Organ Transplantation: Current Indications, Techniques, and Imaging Findings | No | Exclusion based on title and abstract |
| 330 | Banach et al., 2015 | Lipids, blood pressure and kidney update 2014 | No | Exclusion based on title and abstract |
| 331 | Textor et al., 2013 | Percutaneous revascularization for ischemic nephropathy: the past, present, and future | No | Exclusion based on title and abstract |
| 332 | Kwarta et al., 2017 | Cystic Diseases of Childhood: A Review | No | Exclusion based on title and abstract |
| 333 | Seki et al., 2014 | Predicting the development of cardiac allograft vasculopathy | No | Exclusion based on title and abstract |
| 334 | Al-Mufti et al., 2018 | Novel management strategies for medically-refractory vasospasm following aneurysmal subarachnoid hemorrhage | No | Exclusion based on title and abstract |
| 335 | Rangan et al., 2015 | Autosomal Dominant Polycystic Kidney Disease: A Path Forward | No | Exclusion based on title and abstract |
| 336 | Pollack et al., 2013 | Detection and Imaging of Cardiac Allograft Vasculopathy | No | Exclusion based on title and abstract |
| 337 | McGuire et al., 2013 | Pediatric Visceral Transplantation | No | Exclusion based on title and abstract |
| 338 | Stewart et al., 2018 | Prognostic Implications of Left Ventricular Hypertrophy | No | Exclusion based on title and abstract |
| 339 | Toeg et al., 2014 | Atrial Fibrillation Therapies: Lest We Forget Surgery | No | Exclusion based on title and abstract |
| 340 | Kolte et al., 2017 | Interventional Therapies for Heart Failure in Older Adults | No | Exclusion based on title and abstract |
| 341 | Prior et al., 2018 | Neuromuscular diseases associated with Human Immunodeficiency Virus infection | No | Exclusion based on title and abstract |
| 342 | Nguyen et al., 2017 | Anesthetic management of the patient undergoing heart transplantation | No | Exclusion based on title and abstract |
| 343 | Kanai et al., 2013 | New Treatment for Old Disease: Management of Resistant Hypertension by Percutaneous Renal Sympathetic Denervation | No | Different intervention |
| 344 | White et al., 2015 | Renal denervation therapy for hypertension: pathways for moving development forward | No | Different intervention |
| 345 | Azizi et al., 2021 | [Ultrasound renal denervation for hypertension resistant to a triple medication pill (RADIANCE-HTN TRIO): a randomised, multicentre, single-blind, sham-controlled trial](https://www.sciencedirect.com/science/article/pii/S0140673621007881) | No | Duplicates |
| 346 | Verdecchia et al., 2023 | [Catheter-based renal artery denervation: facts and expectations](https://www.sciencedirect.com/science/article/pii/S0953620523002753) | No | Different intervention |
| 347 | Azizi et al., 2020 | [12-Month Results From the Unblinded Phase of the RADIANCE-HTN SOLO Trial of Ultrasound Renal Denervation](https://www.sciencedirect.com/science/article/pii/S1936879820320136) | No | Different methods |
| 348 | Tsiofis et al., 2015 | Catheter-based renal denervation for resistant hypertension: Twenty-four month results of the EnligHTN™ I first-in-human study using a multi-electrode ablation system | No | Different intervention |
| 349 | Bohm et al., 2021 | [Effect of Heart Rate on the Outcome of Renal Denervation in Patients With Uncontrolled Hypertension](https://www.sciencedirect.com/science/article/pii/S0735109721055844) | No | Different intervention, dffferent outcome |
| 350 | Mahfoud et al., 2021 | [Catheter-based alcohol-mediated renal denervation for the treatment of uncontrolled hypertension: design of two sham-controlled, randomized, blinded trials in the absence (TARGET BP OFF-MED) and presence (TARGET BP I) of antihypertensive medications](https://www.sciencedirect.com/science/article/pii/S0002870321001447) | No | Different intervention, different methods |
| 351 | Mauri et al., 2018 | [A multinational clinical approach to assessing the effectiveness of catheter-based ultrasound renal denervation: The RADIANCE-HTN and REQUIRE clinical study designs](https://www.sciencedirect.com/science/article/pii/S0002870317302776) | No | Different intervention |
| 352 | Weber et al., 2020 | [The REDUCE HTN: REINFORCE: Randomized, Sham-Controlled Trial of Bipolar Radiofrequency Renal Denervation for the Treatment of Hypertension](https://www.sciencedirect.com/science/article/pii/S1936879819324124) | No | Different intervention |
| 353 | Skowerski et al., 2016 | Long-term follow-up after radio-frequency catheter-based denervation in patients with resistant hypertension | No | Different intervention |
| 354 | Sas et al., 2018 | [Renal Artery Denervation Due to Refractory Hypertension in a Patient After Kidney Transplantation—3 Years of Observation: A Case Report](https://www.sciencedirect.com/science/article/pii/S0041134518302732) | No | Different study design |
| 355 | Feyz et al., 2020 | [Renal sympathetic denervation in patients with vasospastic angina](https://www.sciencedirect.com/science/article/pii/S1071358123020883) | No | Different intervention, different outcome |
| 356 | Chen et al., 2017 | [Efficacy and Safety of Renal Sympathetic Denervation on Dogs with Pressure Overload-Induced Heart Failure](https://www.sciencedirect.com/science/article/pii/S1443950616315104) | No | Exclusion based on title and abstract |
| 357 | Kandzari er al., 2018 | [Effect of renal denervation on blood pressure in the presence of antihypertensive drugs: 6-month efficacy and safety results from the SPYRAL HTN-ON MED proof-of-concept randomised trial](https://www.sciencedirect.com/science/article/pii/S0140673618309516) | No | Different intervention, different method |
| 358 | Andrew et al., 2015 | Latest Developments in Heart Transplantation: A Review | No | Exclusion based on title and abstract |
| 359 | Ng et al., 2015 | The role of interventional radiology in complications associated with liver transplantation | No | Exclusion based on title and abstract |
| 360 | Olymbias et al., 2018 | Imaging in Heart Transplant Patients | No | Exclusion based on title and abstract |
| 361 | Morin et al., 2016 | The State of the Art: Atrial Fibrillation Epidemiology, Prevention, and Treatment | No | Exclusion based on title and abstract |
| 362 | Higgins et al., 2017 | Surgical Treatment of Heart Failure | No | Exclusion based on title and abstract |
| 363 | Dixon et al., 2013 | The Year in Interventional Cardiology | No | Exclusion based on title and abstract |
| 364 | Gupta et al., 2015 | Imaging in patients after cardiac transplantation and in patients with ventricular assist devices | No | Exclusion based on title and abstract |
| 365 | Stone et al., 2015 | Clinical Trial Design Principles and Endpoint Definitions for Transcatheter Mitral Valve Repair and Replacement: Part 1: Clinical Trial Design Principles: A Consensus Document From the Mitral Valve Academic Research Consortium | No | Exclusion based on title and abstract |
| 366 | Neuzil et al., 2016 | Externally Delivered Focused Ultrasound for Renal Denervation | No | Different methods |
| 367 | Khan et al., 2014 | Renal Denervation Therapy for the Treatment of Resistant Hypertension: A Position Statement by the Canadian Hypertension Education Program | No | Exclusion based on title and abstract |
| 368 | Hu et al., 2015 | Renal sympathetic denervation prevents the development of pulmonary arterial hypertension and cardiac dysfunction in dogs | No | Exclusion based on title and abstract |
| 369 | Fischell et al,.2016 | Transcatheter Alcohol-Mediated Perivascular Renal Denervation With the Peregrine System: First-in-Human Experience | No | Exclusion based on title and abstract |
| 370 | Parvin et al., 2023 | [Sequential afferent and sympathetic renal denervation impact on cardiovascular and renal homeostasis in the male Sprague-Dawley rat](https://www.sciencedirect.com/science/article/pii/S0024320523004022) | No | Exclusion based on title and abstract |
| 371 | Rothman et al., 2020 | [Intravascular Ultrasound Pulmonary Artery Denervation to Treat Pulmonary Arterial Hypertension (TROPHY1): Multicenter, Early Feasibility Study](https://www.sciencedirect.com/science/article/pii/S1936879819326329) | No | Different outcome, different intervention |
| 372 | Kiuchi et al., 2016 | Pulmonary vein isolation alone and combined with renal sympathetic denervation in chronic kidney disease patients with refractory atrial fibrillation | No | Exclusion based on title and abstract |
| 373 | Chrenin et al,, 2017 | [Renal Sympathetic Denervation System via Intraluminal Ultrasonic Ablation: Therapeutic Intravascular Ultrasound Design and Preclinical Evaluation](https://www.sciencedirect.com/science/article/pii/S1051044317301562) | No | Different study design |
| 374 | Sanghvi et al., 2022 | Renal Artery Variations in Patients With Mild-to-Moderate Hypertension From the RADIANCE-HTN SOLO Trial | No | Different intervention |
| 375 | Han et al., 2020 | [The safety of renal denervation as assessed by optical coherence tomography: pre- and post-procedure comparison with multi-electrode ablation catheter in animal experiment](https://www.sciencedirect.com/science/article/pii/S1109966618305256) | No | Exclusion based on title and abstract |
| 376 | Lin et al., 2017 | [Hypotensive effects of renal denervation in spontaneously hypertensive rat based on ultrasonic contrast imaging](https://www.sciencedirect.com/science/article/pii/S0895611117300071) | No | Exclusion based on title and abstract |
| 377 | Qian et al., 2019 | [Transvascular Pacing of Aorticorenal Ganglia Provides a Testable Procedural Endpoint for Renal Artery Denervation](https://www.sciencedirect.com/science/article/pii/S1936879819310891) | No | Different outcome |
| 378 | Delacroix et al., 2018 | [Effects of renal sympathetic denervation on myocardial structure, function and perfusion: A serial CMR study](https://www.sciencedirect.com/science/article/pii/S0021915018301369) | No | Different study design, different outcome |
| 379 | Hoye et al., 2017 | [Endovascular Renal Denervation in End-Stage Kidney Disease Patients: Cardiovascular Protection—A Proof-of-Concept Study](https://www.sciencedirect.com/science/article/pii/S2468024917301110) | No | Different study design |
| 380 | Liu et al., 2016 | [Improvement of cardiac dysfunction by bilateral surgical renal denervation in animals with diabetes induced by high fructose and high fat diet](https://www.sciencedirect.com/science/article/pii/S0168822716000188) | No | Exclusion based on title and abstract |
| 381 | Kiuchi et al., 2016 | [Proof of concept study: Improvement of echocardiographic parameters after renal sympathetic denervation in CKD refractory hypertensive patients](https://www.sciencedirect.com/science/article/pii/S0167527316300663) | No | Exclusion based on title and abstract, concept study |
| 382 | Steinberg et al., 2015 | [Effect of Peripheral Artery Sympathetic Denervation on Muscle Microperfusion and Macroperfusion in an Animal Peripheral Artery Disease Model Using Contrast-Enhanced Ultrasound and Doppler Flow Measurement](https://www.sciencedirect.com/science/article/pii/S1051044315005564) | No | Exclusion based on title and abstract |
| 383 | Chernin et al., 2018 | First-in-Man Experience with a Novel Catheter-Based Renal Denervation System of Ultrasonic Ablation in Patients with Resistant Hypertension | No | Different outcome t |
| 384 | Yamada et al., 2017 | [Renal denervation regulates the atrial arrhythmogenic substrates through reverse structural remodeling in heart failure rabbit model](https://www.sciencedirect.com/science/article/pii/S0167527317311142) | No | Exclusion based on title and abstract |
| 385 | Chen et al., 2013 | [Pulmonary Artery Denervation to Treat Pulmonary Arterial Hypertension: The Single-Center, Prospective, First-in-Man PADN-1 Study (First-in-Man Pulmonary Artery Denervation for Treatment of Pulmonary Artery Hypertension)](https://www.sciencedirect.com/science/article/pii/S0735109713025898) | No | Exclusion based on title and abstract |
| 386 | Mahfoud et al., 2021 | Renal Denervation in High-Risk Patients With Hypertension | No | Different intervention |
| 387 | mahfoud et al., 2022 | [Long-term efficacy and safety of renal denervation in the presence of antihypertensive drugs (SPYRAL HTN-ON MED): a randomised, sham-controlled trial](https://www.sciencedirect.com/science/article/pii/S014067362200455X) | No | Different intervention |
| 388 | Pan et al., 2021 | [Endovascular denervation (EDN): From Hypertension to Non-Hypertension Diseases](https://www.sciencedirect.com/science/article/pii/S209636022100034X) | No | Different intervention |
| 389 | Mahfoud et al., 2020 | [Alcohol-Mediated Renal Denervation Using the Peregrine System Infusion Catheter for Treatment of Hypertension](https://www.sciencedirect.com/science/article/pii/S1936879819323088) | No | Different intervention |
| 390 | Ji et al., 2022 | [Validation of a Novel Renal Denervation System With Cryoablation: A Preclinical Study and Case Series](https://www.sciencedirect.com/science/article/pii/S2452302X21003612) | No | Different study design |
| 391 | Worthley et al., 2017 | [Safety and performance of the second generation EnligHTN™ Renal Denervation System in patients with drug-resistant, uncontrolled hypertension](https://www.sciencedirect.com/science/article/pii/S0021915017301867) | No | Different intervention |
| 392 | Sardar er al., 2019 | [Sham-Controlled Randomized Trials of Catheter-Based Renal Denervation in Patients With Hypertension](https://www.sciencedirect.com/science/article/pii/S0735109719304917) | No | Different intervention |
| 393 | Moriarty et al., 2016 | Renal Denervation: A Novel Therapy at the Crossroads of Imaging, Intervention, and Innovation | No | Different study design, diifferent intervention |
| 394 | Davies et al., 2013 | First-in-man safety evaluation of renal denervation for chronic systolic heart failure: Primary outcome from REACH-Pilot study | No | Different intervention |
| 395 | Patel et al., 2013 | [A cross-sectional imaging study to identify organs at risk of thermal injury during renal artery sympathetic denervation](https://www.sciencedirect.com/science/article/pii/S0167527315300012) | No | Different study deisgn, different outcome |
| 396 | Ott et al,. 2014 | Improvement of albuminuria after renal denervation | No | Different intervention, different outcome |
| 397 | Bernandi et al., 2013 | Percutaneous Sympathetic Renal Denervation | No | Different intervention |
| 398 | Ewen et al., 2016 | Anatomical and procedural determinants of catheter-based renal denervation | No | Different intervention, different outcome |
| 399 | Evranos et al., 2016 | [Role of Adjuvant Renal Sympathetic Denervation in the Treatment of Ventricular Arrhythmias](https://www.sciencedirect.com/science/article/pii/S0002914916312450) | No | Exclusion based on title and abstract |
| 400 | Schlaich et al,, 2013 | [Feasibility of catheter-based renal nerve ablation and effects on sympathetic nerve activity and blood pressure in patients with end-stage renal disease](https://www.sciencedirect.com/science/article/pii/S0167527313002787) | No | Exclusion based on title and abstract |
| 401 | Nogonaki et al., 2016 | [Low-frequency and very low-intensity ultrasound decreases blood pressure in hypertensive subjects with type 2 diabetes](https://www.sciencedirect.com/science/article/pii/S0167527316307677) | No | Exclusion based on title and abstract |
| 402 | Vemullapalii et al., 2014 | [Proceedings from Duke Resistant Hypertension Think Tank](https://www.sciencedirect.com/science/article/pii/S0002870314000891) | No | Exclusion based on title and abstract |
| 403 | Verdalles et al., 2016 | [Prevalence and characteristics of patients with resistant hypertension and chronic kidney disease](https://www.sciencedirect.com/science/article/pii/S2013251416301171) | No | Exclusion based on title and abstract |
| 404 | Carnevale et al., 2014 | [The Angiogenic Factor PlGF Mediates a Neuroimmune Interaction in the Spleen to Allow the Onset of Hypertension](https://www.sciencedirect.com/science/article/pii/S1074761314003975) | No | Exclusion based on title and abstract |
| 405 | Narkiewicz., 2016 | [Unilateral Carotid Body Resection in Resistant Hypertension: A Safety and Feasibility Trial](https://www.sciencedirect.com/science/article/pii/S2452302X1630064X) | No | Exclusion based on title and abstract |
| 406 | El-din et al., 2016 | [Left ventricular hypertrophy in controlled hypertension: Is blood pressure variability blamed?](https://www.sciencedirect.com/science/article/pii/S1110260815000241) | No | Exclusion based on title and abstract |
| 407 | Zhang et al,. 2019 | Pulmonary Artery Denervation Significantly Increases 6-Min Walk Distance for Patients With Combined Pre- and Post-Capillary Pulmonary Hypertension Associated With Left Heart Failure: The PADN-5 Study | No | Different outcome |
| 408 | Gao et al., 2017 | [Effects of percutaneous renal sympathetic denervation on cardiac function and exercise tolerance in patients with chronic heart failure](https://www.sciencedirect.com/science/article/pii/S2174204916302719) | No | Different outcome |
| 409 | Zheng et al., 2023 | Pulmonary Artery Denervation Inhibits Left Stellate Ganglion Stimulation-Induced Ventricular Arrhythmias Originating From the RVOT | No | Exclusion based on title and abstract |
| 410 | Rosendorff et al., 2015 | [Treatment of hypertension in patients with coronary artery disease: A scientific statement from the American Heart Association, American College of Cardiology, and American Society of Hypertension](https://www.sciencedirect.com/science/article/pii/S1933171115000972) | No | Exclusion based on title and abstract |
| 411 | Modolo et al,m 2015 | [Refractory and resistant hypertension: characteristics and differences observed in a specialized clinic](https://www.sciencedirect.com/science/article/pii/S193317111500100X) | No | Exclusion based on title and abstract |
| 412 | Keegan et al., 2015 | [Inter-study reproducibility of interleaved spiral phase velocity mapping of renal artery haemodynamics](https://www.sciencedirect.com/science/article/pii/S1097664723008335) | No | Exclusion based on title and abstract |
| 413 | Coats et al., 2013 | [Hypertensive subjects with type-2 diabetes, the sympathetic nervous system, and treatment implications](https://www.sciencedirect.com/science/article/pii/S0167527314008869) | No | Exclusion based on title and abstract |
| 414 | Castel et al., 2013 | [Long-term prognostic value of elevated heart rate one year after heart transplantation](https://www.sciencedirect.com/science/article/pii/S0167527312017330) | No | Exclusion based on title and abstract |
| 415 | Georgiopoulos et al., 2016 | [Metabolic syndrome, independent of its components, affects adversely cardiovascular morbidity in essential hypertensives](https://www.sciencedirect.com/science/article/pii/S0021915015301830) | No | Exclusion based on title and abstract |
| 416 | Zheng et al., 2023 | [Pulmonary Artery Denervation Inhibits Left Stellate Ganglion Stimulation-Induced Ventricular Arrhythmias Originating From the RVOT](https://www.sciencedirect.com/science/article/pii/S2405500X23001056) | No | Exclusion based on title and abstract |
| 417 | Lui et al., 2023 | [Characterizing Hypertension Specialist Care in Canada: A National Survey](https://www.sciencedirect.com/science/article/pii/S2589790X23002457) | No | Exclusion based on title and abstract |
| 418 | Sato et al., 2021 | [Comprehensive Assessment of Human Accessory Renal Artery Periarterial Renal Sympathetic Nerve Distribution](https://www.sciencedirect.com/science/article/pii/S1936879820319920) | No | Exclusion based on title and abstract |
| 419 | Khalid et al., 2019 | [Overview of the 2018 US Food and Drug Administration Circulatory System Devices Panel Meeting on Device-Based Therapies for hypertension](https://www.sciencedirect.com/science/article/pii/S1553838919304282) | No | Exclusion based on title and abstract |
| 420 |  |  | No | Exclusion based on title and abstract |
| 421 |  |  | No | Exclusion based on title and abstract |
| 422 |  |  | No | Exclusion based on title and abstract |
| 423 | Pekarskiy et al., 2022 | [Durable strong efficacy and favorable long-term renal safety of the anatomically optimized distal renal denervation according to the 3 year follow-up extension of the double-blind randomized controlled trial](https://www.sciencedirect.com/science/article/pii/S2405844022000354) | No | Different outcome |
| 424 | Engholm et al., 2018 | [Effects of renal denervation on coronary flow reserve and forearm dilation capacity in patients with treatment-resistant hypertension. A randomized, double-blinded, sham-controlled clinical trial](https://www.sciencedirect.com/science/article/pii/S0167527317341864) | No | Different outcome |
| 425 | Cai et al., 2019 | [Noninvasive Stereotactic Radiotherapy for Renal Denervation in a Swine Model](https://www.sciencedirect.com/science/article/pii/S0735109719361522) | No | Exclusion based on title and abstract |
| 426 | Steinberg et al., 2014 | [Very long-term outcome after initially successful catheter ablation of atrial fibrillation](https://www.sciencedirect.com/science/article/pii/S1547527114001234) | No | Exclusion based on title and abstract |
| 427 | Sadaghianloo et al., 2016 | [Radial artery deviation and reimplantation inhibits venous juxta-anastomotic stenosis and increases primary patency of radial-cephalic fistulas for hemodialysis](https://www.sciencedirect.com/science/article/pii/S074152141630163X) | No | Exclusion based on title and abstract |
| 428 | Xia et al., 2022 | [Cardiac Spinal Afferent Denervation Attenuates Renal Dysfunction in Rats With Cardiorenal Syndrome Type 2](https://www.sciencedirect.com/science/article/pii/S2452302X22000456) | No | Exclusion based on title and abstract |
| 429 | Dena et al., 2018 | [Role of MDCT renal angiography in determining the anatomical eligibility for renal sympathetic denervation in resistant hypertensive patients](https://www.sciencedirect.com/science/article/pii/S0378603X17302048) | No | Exclusion based on title and abstract |
| 430 | Hori et al., 2021 | [Aorticorenal ganglion as a novel target for renal neuromodulation](https://www.sciencedirect.com/science/article/pii/S1547527121018051) | No | Exclusion based on title and abstract |
| 431 | Khan et al., 2019 | [Is there still a role for renal artery stenting in the management of renovascular hypertension – A single-center experience and where do we stand?](https://www.sciencedirect.com/science/article/pii/S1553838918302549) | No | Exclusion based on title and abstract |
| 432 | Chen et al., 2019 | [Prenatal cold exposure causes hypertension in offspring by hyperactivity of the sympathetic nervous system](https://www.sciencedirect.com/science/article/pii/S147087361900125X) | No | Exclusion based on title and abstract |
| 433 | Ditac et al., 2023 | [Therapeutic Ultrasound Applications in Cardiovascular Diseases: A Review](https://www.sciencedirect.com/science/article/pii/S1959031822000781) | No | Exclusion based on title and abstract |
| 434 | Cao et al., 2018 | A renal-cerebral-peripheral sympathetic reflex mediates insulin resistance in chronic kidney disease | No | Exclusion based on title and abstract |
| 435 | Badr et al., 2019 | [Relation of neuropeptide Y gene expression and genotyping with hypertension in chronic kidney disease](https://www.sciencedirect.com/science/article/pii/S2405580819301384) | No | Exclusion based on title and abstract |
| 436 | Gao et al.,2017 | [Effects of percutaneous renal sympathetic denervation on cardiac function and exercise tolerance in patients with chronic heart failure](https://www.sciencedirect.com/science/article/pii/S2174204916302719) | No | Different outcome |
| 437 | Roy et al., 2023 | [Preoperative assessment of inferior vena cava collapsibility index by ultrasound is not a reliable predictor of post-spinal anesthesia hypotension](https://www.sciencedirect.com/science/article/pii/S0104001422000513) | No | Exclusion based on title and abstract |
| 438 | Baratta et al., 2023 | [Right Heart Adaptation to Exercise in Pulmonary Hypertension: An Invasive Hemodynamic Study](https://www.sciencedirect.com/science/article/pii/S1071916423001513) | No | Exclusion based on title and abstract |
| 439 | Liga et al., 2022 | Cardiac sympathetic dysfunction in left ventricular hypertrophy caused by arterial hypertension and degenerative aortic stenosis | No | Exclusion based on title and abstract |
| 440 | Marsico et al., 2021 | [Renal function and cardiac adrenergic impairment in patients affected by heart failure](https://www.sciencedirect.com/science/article/pii/S1071358123015660) | No | Exclusion based on title and abstract |
| 441 | Ars et al., 2022 | [Consensus document on autosomal dominant polycystic kindey disease from the Spanish Working Group on Inherited Kindey Diseases. Review 2020](https://www.sciencedirect.com/science/article/pii/S2013251422001213) | No | Exclusion based on title and abstract |
| 442 | Elijovich et al., 2020 | [Hypothesis: Unrecognized actions of ENaC blockade in improving refractory-resistant hypertension and residual cardiovascular risk](https://www.sciencedirect.com/science/article/pii/S2590086220300252) | No | Exclusion based on title and abstract |
| 443 | Sehgal et al.,2020 | [Fetal Growth Restriction and Hypertension in the Offspring: Mechanistic Links and Therapeutic Directions](https://www.sciencedirect.com/science/article/pii/S0022347620306144) | No | Exclusion based on title and abstract |
| 444 | Chinushi et al., 2020 | [Enhanced arrhythmogenic potential induced by renal autonomic nerve stimulation: Role of renal artery catheter ablation](https://www.sciencedirect.com/science/article/pii/S1547527119306708) | No | Different intervention, different outcome |
| 445 | Bradfield et al., 2018 | [Mechanisms and management of refractory ventricular arrhythmias in the age of autonomic modulation](https://www.sciencedirect.com/science/article/pii/S1547527118301127) | No | Exclusion based on title and abstract |
| 446 | Spinelli et al., 2021 | [Focal reduction in left ventricular 123I-metaiodobenzylguanidine uptake and impairment in systolic function in patients with Anderson-Fabry disease](https://www.sciencedirect.com/science/article/pii/S1071358123008164) | No | Exclusion based on title and abstract |
| 447 | Min et al., 2021 | [Clinical significance of heart rate variability for the monitoring of cardiac autonomic neuropathy in end-stage renal disease patients](https://www.sciencedirect.com/science/article/pii/S0939475321001381) | No | Exclusion based on title and abstract |
| 448 | Sam et al., 2023 | [Outcomes among heart transplant recipients following acute coronary syndrome: A nationwide population based study](https://www.sciencedirect.com/science/article/pii/S0167527322012670) | No | Exclusion based on title and abstract |
| 449 | Bencivega et al., 2021 | [Impact of the number of comorbidities on cardiac sympathetic derangement in patients with reduced ejection fraction heart failure](https://www.sciencedirect.com/science/article/pii/S0953620521000108) | No | Exclusion based on title and abstract |
| 450 | Honda et al., 2023 | [Effects of olmesartan and amlodipine on blood pressure, endothelial function, and vascular inflammation](https://www.sciencedirect.com/science/article/pii/S1071358124000618) | No | Exclusion based on title and abstract |
| 451 | Badr et el., 2019 | [Relation of neuropeptide Y gene expression and genotyping with hypertension in chronic kidney disease](https://www.sciencedirect.com/science/article/pii/S2405580819301384) | No | Exclusion based on title and abstract |
| 452 | Roy et al., 2023 | [Preoperative assessment of inferior vena cava collapsibility index by ultrasound is not a reliable predictor of post-spinal anesthesia hypotension](https://www.sciencedirect.com/science/article/pii/S0104001422000513) | No | Exclusion based on title and abstract |
| 453 | Baratto et al., 2023 | [Preoperative assessment of inferior vena cava collapsibility index by ultrasound is not a reliable predictor of post-spinal anesthesia hypotension](https://www.sciencedirect.com/science/article/pii/S0104001422000513) | No | Exclusion based on title and abstract |
| 454 | Marsico., 2021 | [Renal function and cardiac adrenergic impairment in patients affected by heart failure](https://www.sciencedirect.com/science/article/pii/S1071358123015660) | No | Exclusion based on title and abstract |
| 455 | Elijovich et al., 2020 | [Hypothesis: Unrecognized actions of ENaC blockade in improving refractory-resistant hypertension and residual cardiovascular risk](https://www.sciencedirect.com/science/article/pii/S2590086220300252) | No | Exclusion based on title and abstract |
| 456 | Sehgal et al., 2020 | [Fetal Growth Restriction and Hypertension in the Offspring: Mechanistic Links and Therapeutic Directions](https://www.sciencedirect.com/science/article/pii/S0022347620306144) | No | Exclusion based on title and abstract |
| 457 | Chinushi et al., 2020 | [Enhanced arrhythmogenic potential induced by renal autonomic nerve stimulation: Role of renal artery catheter ablation](https://www.sciencedirect.com/science/article/pii/S1547527119306708) | No | Exclusion based on title and abstract |
| 458 | Bradfield et al., 2018 | [Mechanisms and management of refractory ventricular arrhythmias in the age of autonomic modulation](https://www.sciencedirect.com/science/article/pii/S1547527118301127) | No | Exclusion based on title and abstract |
| 459 | Min et al., 2021 | [Clinical significance of heart rate variability for the monitoring of cardiac autonomic neuropathy in end-stage renal disease patients](https://www.sciencedirect.com/science/article/pii/S0939475321001381) | No | Exclusion based on title and abstract |
| 460 | Xue et al., 2021 | [Downregulating the P2X3 receptor in the carotid body to reduce blood pressure via acoustic gene delivery in canines](https://www.sciencedirect.com/science/article/pii/S1931524420301456) | No | Exclusion based on title and abstract |
| 461 | Bencivenga et al., 2021 | [Impact of the number of comorbidities on cardiac sympathetic derangement in patients with reduced ejection fraction heart failure](https://www.sciencedirect.com/science/article/pii/S0953620521000108) | No | Exclusion based on title and abstract |
| 462 | Ram et al., 2023 | [Outcomes among heart transplant recipients following acute coronary syndrome: A nationwide population based study](https://www.sciencedirect.com/science/article/pii/S0167527322012670) | No | Exclusion based on title and abstract |
| 463 | Honda et al., 2023 | [Effects of olmesartan and amlodipine on blood pressure, endothelial function, and vascular inflammation](https://www.sciencedirect.com/science/article/pii/S1071358124000618) | No | Exclusion based on title and abstract |
| 464 | Park et al., 2023 | [Invasive aortic pulse pressure is linked to cardiac allograft vasculopathy after heart transplantation](https://www.sciencedirect.com/science/article/pii/S0167527322016709) | No | Exclusion based on title and abstract |
| 465 | Irani et al., 2023 | [Society for Maternal-Fetal Medicine Consult Series #66: Prepregnancy evaluation and pregnancy management of patients with solid organ transplants](https://www.sciencedirect.com/science/article/pii/S0002937823002533) | No | Exclusion based on title and abstract |
| 466 | Kaminska et al., 2018 | [Issues of Immunological and Hemodynamic Monitoring Before and During Kidney Transplantation in Sensitized Heart Transplant Recipient](https://www.sciencedirect.com/science/article/pii/S004113451830352X) | No | Exclusion based on title and abstract |
| 467 | Cao et al., 2019 | [Adipocytes initiate an adipose-cerebral-peripheral sympathetic reflex to induce insulin resistance during high-fat feeding](https://www.sciencedirect.com/science/article/pii/S1470873619000838) | No | Exclusion based on title and abstract |
| 468 | Rossi et al., 2021 | [The cardiovascular consequences of hyperaldosteronism](https://www.sciencedirect.com/science/article/pii/S0003426620300299) | No | Exclusion based on title and abstract |
| 469 | Ting et al., 2023 | [An alternative lower tract approach to ectopic duplex system ureteroceles feasible in young children](https://www.sciencedirect.com/science/article/pii/S1477513122004089) | No | Exclusion based on title and abstract |
| 470 | Tanaka et al., 2023 | [Impact of myocardial bridging on coronary artery plaque formation and long-term mortality after heart transplantation](https://www.sciencedirect.com/science/article/pii/S0167527323003431) | No | Exclusion based on title and abstract |
| 471 | Kahlberg et al., 2021 | [Propensity-Matched Comparison for Carotid Artery Stenting in Primary Stenosis Versus after Carotid Endarterectomy Restenosis](https://www.sciencedirect.com/science/article/pii/S0890509620305653) | No | Exclusion based on title and abstract |
| 472 | Lee et al., 2022 | [Characteristics of surgically treated Guyon canal syndrome: A multicenter retrospective study](https://www.sciencedirect.com/science/article/pii/S1748681522002479) | No | Exclusion based on title and abstract |
| 473 | Bonito et al., 2020 | [Elevated blood pressure, cardiometabolic risk and target organ damage in youth with overweight and obesity](https://www.sciencedirect.com/science/article/pii/S0939475320302179) | No | Exclusion based on title and abstract |
| 474 | Penner et al., 2021 | [6-month multidisciplinary follow-up and outcomes of patients with paediatric inflammatory multisystem syndrome (PIMS-TS) at a UK tertiary paediatric hospital: a retrospective cohort study](https://www.sciencedirect.com/science/article/pii/S2352464221001383) | No | Exclusion based on title and abstract |
| 475 | Tchana-Sato et al., 2022 | [Abdominal Aortic Aneurysm in Heart Transplant Recipients: New Insights from a 30-year Experience at a Single Center](https://www.sciencedirect.com/science/article/pii/S0890509622003119) | No | Exclusion based on title and abstract |
| 476 | Patel et al., 2019 | [Diagnosis and Treatment of Renovascular Disease in Children](https://www.sciencedirect.com/science/article/pii/S0037198X19300446) | No | Exclusion based on title and abstract |
| 477 | Sibai et al., 2023 | [A Narrative Review of the Evaluation and Management of Liver Transplant Complications in the Emergency Department](https://www.sciencedirect.com/science/article/pii/S0736467923000690) | No | Exclusion based on title and abstract |
| 478 | Cortigiani et al., 2021 | [Prognostic Value of Heart Rate Reserve during Dipyridamole Stress Echocardiography in Patients With Abnormal Chronotropic Response to Exercise](https://www.sciencedirect.com/science/article/pii/S0002914921005300) | No | Exclusion based on title and abstract |
| 479 | Cho et al., 2021 | [Effect of Extracorporeal Shock Wave Therapy on Muscle Mass and Function in Patients Undergoing Maintenance Hemodialysis: A Randomized Controlled Pilot Study](https://www.sciencedirect.com/science/article/pii/S0301562921003331) | No | Exclusion based on title and abstract |
| 480 | Vassamseti et al., 2018 | [Sympathetic Neuronal Activation Triggers Myeloid Progenitor Proliferation and Differentiation](https://www.sciencedirect.com/science/article/pii/S1074761318302401) | No | Exclusion based on title and abstract |
| 481 | Enezate et al., 2018 | [Outcomes of Acute Myocardial Infarction in Heart Transplant Recipients](https://www.sciencedirect.com/science/article/pii/S0002914918317661) | No | Exclusion based on title and abstract |
| 482 | Keer et al., 2019 | [Long-term outcome of cardiac allograft vasculopathy: Importance of the International Society for Heart and Lung Transplantation angiographic grading scale](https://www.sciencedirect.com/science/article/pii/S1053249819316237) | No | Exclusion based on title and abstract |
| 483 | Jiang et al., 2022 | [Coronary artery disease in adults with anomalous aortic origin of a coronary artery](https://www.sciencedirect.com/science/article/pii/S266627362200184X) | No | Exclusion based on title and abstract |
| 484 | Velleca et al., 2023 | [The International Society for Heart and Lung Transplantation (ISHLT) guidelines for the care of heart transplant recipients](https://www.sciencedirect.com/science/article/pii/S1053249822021854) | No | Exclusion based on title and abstract |
| 485 | Cao et al., 2021 | [Bone Marrow Edema Syndrome of the Foot Treated with Extracorporeal Shock Wave Therapy: A Retrospective Case Series](https://www.sciencedirect.com/science/article/pii/S1067251620303914) | No | Exclusion based on title and abstract |
| 486 | Darby et al., 2020 | Cardiorespiratory consequences of intrauterine growth restriction: Influence of timing, severity and duration of hypoxaemia | No | Exclusion based on title and abstract |
