# Supplementary material for: Ultrasound renal denervation in hypertensive patients: A systematic review and meta-analysis
Source: PLoS One. 2025 Jan 15;20(1):e0311191. doi: 10.1371/journal.pone.0311191 (PMC11734901; doi:10.1371/journal.pone.0311191)
Supplement: Attachment — Submitted filename: Response to reviewer_plosone 20240820 (1).docx [file pone.0311191.s004.docx]

Dr. Emily Chenette

Editor-in-Chief

Journal of PLOS One

Dear Dr. Emily Chenette,

I am writing to submit a revised version of our manuscript entitled “Ultrasound Renal Denervation in Hypertensive Patients: A Systematic Review and Meta-Analysis” to PLOS One. We greatly appreciate the constructive feedback provided by the reviewers and have made significant revisions to the manuscript to address their concerns.

We have carefully considered all the reviewers' comments and made several changes accordingly. We have elaborated in narration text regarding the characteristics of each study in more detail in our main manuscript. The changes and additional data have been outlined in the attached response to reviewers. Additionally, we have included a highlighted version of the manuscript to indicate the revisions made.

These changes have significantly improved the manuscript and addressed the reviewers' concerns. We hope you will find this revised version suitable for publication in PLOS One.

Sincerely,

Maulana A. Empitu, M.D., Ph.D.

Faculty of Medicine, Airlangga University, Surabaya, Indonesia

Email: [maulana.antiyan@fk.unair.ac.id](mailto:maulana.antiyan@fk.unair.ac.id)

**RESPONSE TO REVIEWER QUESTION**

**EDITOR COMMENTS:**

When submitting your revision, we need you to address these additional requirements.

1. Whilst the authors have responded to most comments appropriately, I am not persuaded that they addressed the issue about adequately summarizing the nature of the different populations. There is still no narrative text in the results section and there should be. I can see the supplemental table that reviewers say includes the description of the population description to judge if adequate.

Thank you for your thorough review and for pointing out the need for a more detailed narrative regarding the characteristics of the different populations in our study. We have included a more comprehensive narrative in the results section of our main manuscript (page 6-9). This narrative text elaborates on the characteristics of each study population in greater detail, addressing the diversity and specific attributes of the populations involved. Additionally, we have ensured that this information is now directly accessible in the main text and not only the supplemental tables (page 6-9). We appreciate your advice in improving the clarity and comprehensiveness of our manuscript.
